# Development of a Highly Durable Photocatalytic CO_2_ Reduction Using a Mn-Complex Catalyst: Application of Selective Photosplitting of a Mn(0)–Mn(0) Bond

**DOI:** 10.1021/jacs.4c18366

**Published:** 2025-02-10

**Authors:** Hiroki Koizumi, Yusuke Tamaki, Kei Kamogawa, Marco Nicaso, Yutaka Suzuki, Yasuomi Yamazaki, Hiroyuki Takeda, Osamu Ishitani

**Affiliations:** † National Institute of Advanced Industrial Science and Technology (AIST), Tsukuba Central 5, 1-1-1 Higashi, Tsukuba, Ibaraki 305-8565, Japan; ‡ National Institute of Advanced Industrial Science and Technology (AIST), 4-2-1 Nigatake, Miyagino, Sendai, Miyagi 983-8551, Japan; § Department of Chemistry, School of Science, Tokyo Institute of Technology, O-okayama 2-12-1-NE-1, Meguro, Tokyo 152-8550, Japan; ∥ Department of Applied Chemistry, School of Engineering, The University of Tokyo, 7-3-1 Hongo, Bunkyo, Tokyo 113-8656, Japan; ⊥ Division of Molecular Science, Faculty of Science and Technology, Gunma University, 1-5-1 Tenjin, Kiryu, Gunma 376-8515, Japan; # Department of Chemistry, Graduate School of Advanced Science and Engineering, Hiroshima University, 1-3-1 Kagamiyama, Higashi-Hiroshima, Hiroshima 739-8526, Japan

## Abstract

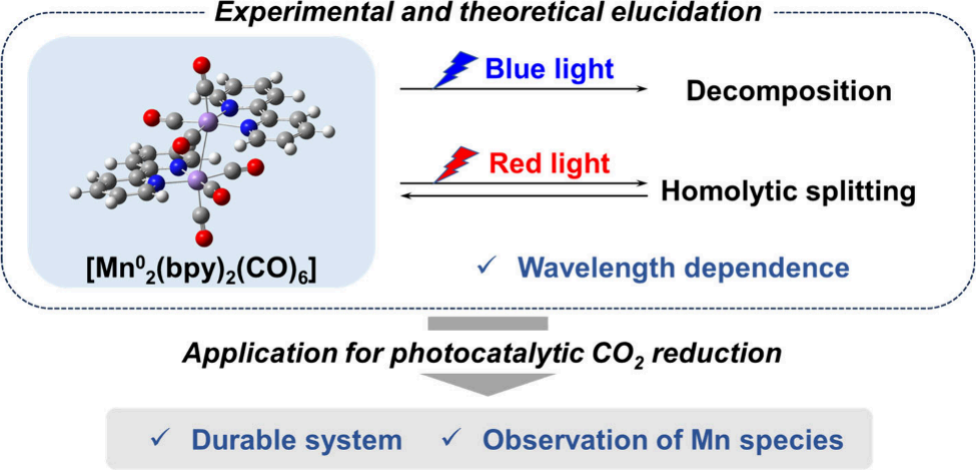

*fac*-[Mn^I^(diimine)(CO)_3_(L)]^0/+^ has attracted significant attention as a catalyst for the photocatalytic reduction of CO_2_. However, in such photocatalytic systems, the photoexcitation of Mn complexes and reaction intermediates induces their decomposition, which lowers the durability of these systems. In this study, we clarified the primary process whereby the Mn complex catalyst decomposes during the photocatalytic reaction. Based thereupon, we successfully constructed a highly durable photocatalytic system, of which the turnover number of formate (TON_HCOO–_) exceeded 1700 when *fac*-[Mn^I^(bpy)(CO)_3_((OC(O)OC_2_H_5_N(C_2_H_5_OH)_2_) (**Mn-CO**_**2**_**-TEOA**) as the catalyst, [Os^II^(4,4′-dimethyl-bpy)(5,5′-dimethyl-bpy)_2_]^2+^ (**Os**) as the photosensitizer, and 1,3-dimethyl-2-phenyl-2,3-dihydro-1H-benzo[d]imidazole (**BIH**) as the reductant were used in conjunction with irradiation at λ_ex_ ≥ 620 nm. In contrast, for the same photocatalytic system, irradiation at λ_ex_ ≥ 480 nm lowered the TON_HCOO–_ to less than 60. The significant difference in the durability of the photocatalytic system arises from the dependence of the Mn(0)–Mn(0) dimer [Mn^0^_2_(bpy)_2_(CO)_6_] (**Dim-Mn**), an intermediate produced during the photocatalytic reaction, on the wavelength of the irradiated light for its photoreactivity. That is, the irradiation of **Dim-Mn** at λ_ex_ ≥ 620 nm selectively induces splitting of the Mn–Mn bond to produce [Mn^0^(bpy)(CO)_3_] (**Mn**^•^) and, contrary to this, splitting of the Mn(0)–CO bonds and further decomposition processes are induced by irradiation at λ_ex_ ≥ 480 nm.

## Introduction

The development of photocatalytic redox systems for the conversion of CO_2_ to high-energy materials is a promising strategy to address global warming—caused by the increase in the atmospheric CO_2_ concentration—and the unavoidable future shortage of carbon and energy resources.^1,2^ In particular, several studies have focused on photocatalytic systems that consist of two types of metal complexes: as photosensitizers that photochemically initiate the transfer of one electron from the reductant to the catalyst and as catalysts that accept electrons from the photosensitizers and reduce CO_2_. In recent years, not only have heavy transition metal ions such as Ru(II), Re(I), Ir(III) and Rh(II)^3−10^ been used as the central metal ion in catalysts, but earth-abundant first-row transition metal ions such as Mn(I), Co(III), Ni(II) and Fe(II)^11−20^ have also been frequently used to develop catalysts for the photocatalytic reduction of CO_2_.

Mn(I) tricarbonyl complexes have attracted attention as catalysts since the electrochemical reduction of CO_2_ to produce CO was reported by Deronzier and co-workers in 2011.^21^ These complexes exhibited a lower overpotential for CO_2_ reduction (compared to the well-studied Re(I) complexes with a similar structure) and high catalytic activity for CO_2_ reduction. Electrochemical CO_2_ reduction systems based on Mn(I) complexes bearing various ligands have been developed in recent years.^22−32^ In comparison to the electrochemical reduction of CO_2_, few reports on the photocatalytic reduction of CO_2_ using Mn(I) catalysts in homogeneous systems have appeared because the Mn carbonyl complexes frequently decompose during the photocatalytic reactions.^33−37^ Mn(I) tricarbonyl complexes are well-known for their ability to release CO upon exposure to light.^38^ Among these Mn(I) complexes, typical Mn(I) catalysts for the reduction of CO_2_ such as *fac*-[Mn^I^(bpy)(CO)_3_X] (bpy = 2,2-bipyridine, X = halogen) not only release CO but also lead to the formation of the Mn(0) dimer [Mn^0^_2_(bpy)_2_(CO)_6_] (**Dim-Mn**), after releasing the X^–^ ligand from the one-electron reduced species of the starting complex.^39−44^ Because *fac*-[Mn^I^(bpy)(CO)_3_X] exhibits UV–vis absorption up to ∼500 nm, decomposition of *fac*-[Mn^I^(bpy)(CO)_3_X] through direct excitation can be suppressed by using certain photosensitizers with longer-wavelength absorption, such as Ru(II) trisdiimine complexes. In the initial stage of the photocatalytic reduction of CO_2_, **Dim-Mn** rapidly forms and exhibits strong absorption across the entire visible wavelength region. We reported that the excitation of **Dim-Mn** with light (λ_ex_ = 480 nm) induces the decomposition of **Dim-Mn** with the release of CO to form Mn-paramagnetic species.^45^ Hartl and co-workers also reported that decomposition with the release of CO and homolytic cleavage of **Dim-Mn** simultaneously occurred during irradiation at λ_ex_ = 405 nm in their photoassisted electrocatalytic CO_2_ reduction system.^46^ Meanwhile, Fujita and Muckerman reported photolytic cleavage and reformation of a Re(0) dimer, [Re^0^_2_(bpy)_2_(CO)_6_], of which the structure and absorption bands in the UV–vis region resembled those of **Dim-Mn**.^47^ The homolytic cleavage of [Re^0^_2_(bpy)_2_(CO)_6_] was induced by photolysis (λ_ex_ ≥ 380 nm) to produce the Re(0) species, [Re^0^(bpy)(CO)_3_(THF)], in THF, which slowly dimerized to [Re_2_(bpy)_2_(CO)_6_] (rate constant of dimerization *k*_d_ = 11 ± 4 M^–1^ s^–1^). During this homolytic cleavage and subsequent dimerization, [Re^0^_2_(bpy)_2_(CO)_6_] was almost quantitatively recovered (>95%). **Dim-Mn** accumulates and decomposes more easily than [Re^0^_2_(bpy)_2_(CO)_6_] during the photocatalytic reduction of CO_2_. Thus, considering the decomposition of the Mn catalyst, **Dim-Mn** is a key intermediate among the Mn species formed during the photocatalytic reaction. However, as the photochemical reactivity of **Dim-Mn** has not been systematically studied, general guidelines for the development of efficient and durable photocatalytic systems using Mn(I) complexes as catalysts are not yet available.

**Scheme 1:**
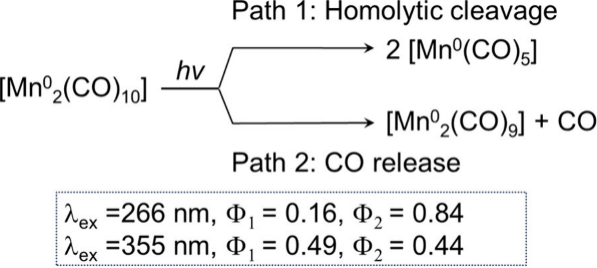
Reported Photochemical Reactivities of [Mn^0^_2_(CO)_10_] Φ_n_: quantum yield.

In contrast to **Dim-Mn**, the photochemical properties of [Mn^0^_2_(diimine)(CO)_8_] and [Mn^0^_2_(CO)_10_], each with an Mn(0)–Mn(0) bond and Mn(0)–CO bonds, similar to **Dim-Mn**, have been actively studied.^48−56^ These Mn(0) complexes exhibit unique photoreactivity: for instance, irradiation of [Mn^0^_2_(CO)_10_] at different wavelengths in cyclohexane induces a change in the ratio between the quantum yields of the two different reactions, i.e., irradiation at λ_ex_ = 266 nm mainly releases a CO ligand and, contrary to this, irradiation at λ_ex_ = 355 nm more selectively induces homolytic cleavage of the Mn(0)–Mn(0) bond (Scheme 1).^52^ Therefore, both [Mn^0^_2_(diimine)(CO)_8_] and [Mn^0^_2_(CO)_10_] do not follow Kasha’s rule.^57^ This led us to hypothesize that if the photochemical reactivity of **Dim-Mn** could be shown to be similar to those of the above-mentioned two Mn(0) complexes, it might be possible to control the durability of the Mn(I) complex in photocatalytic systems by selecting the wavelength of the light used for irradiation.

**Figure 1:**
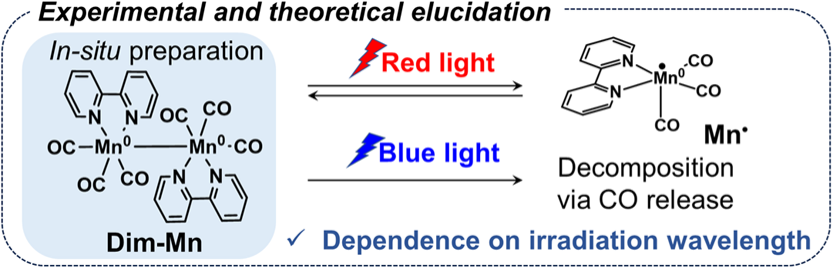
Dependence of the photochemical reactivity of **Dim-Mn** on the wavelength of the light used for irradiation.

Herein, we elucidate the drastic change in the photochemical reactivity of **Dim-Mn** depending on the irradiation wavelength (Figure 1) and demonstrate its successful application to construct a highly durable photocatalytic system with an Mn (I) complex as the catalyst and an Os(II) complex as the photosensitizer. We also report the mechanism by which this durable photocatalytic reaction system operates.

## Results and Discussion

### Photochemical Reactions of Dim-Mn

Initially, we investigated the photochemical reactivity of **Dim-Mn** using two monochromatic lights at λ_ex_ = 480 and 650 nm. To synthesize **Dim-Mn** in a *N,N*-dimethylacetoamide (DMA)-triethanolamine (TEOA) (5:1 v/v) mixed solution, [Co(1,2,3,4,5-pentamethylcyclopentadienyl)_2_] (CoCp*) was used as the one-electron donor as reported^58^ because its redox potential (*E*_1/2_(Co^II^/Co^III^) = –1.87 V vs Fc/Fc^+^) is sufficiently negative to reduce *fac*-[Mn^I^(bpy)(CO)_3_Br] (**Mn-Br**) and, additionally, the reactivity of CoCp* is relatively low except for the donation of an electron. Two DMA-TEOA solutions containing **Mn-Br** or CoCp* were mixed in a glovebox filled with Ar to obtain **Dim-Mn**. The FT-IR and UV–vis absorption spectra of the produced solution corresponded with those of **Dim-Mn** in previous reports.^44^ The photochemical reactions of *in situ* prepared **Dim-Mn** were investigated by irradiating the solution with monochromic light at two different wavelengths (λ_ex_ = 480 and 650 nm) using *in situ* UV–vis absorption spectroscopy.

**Figure 2:**
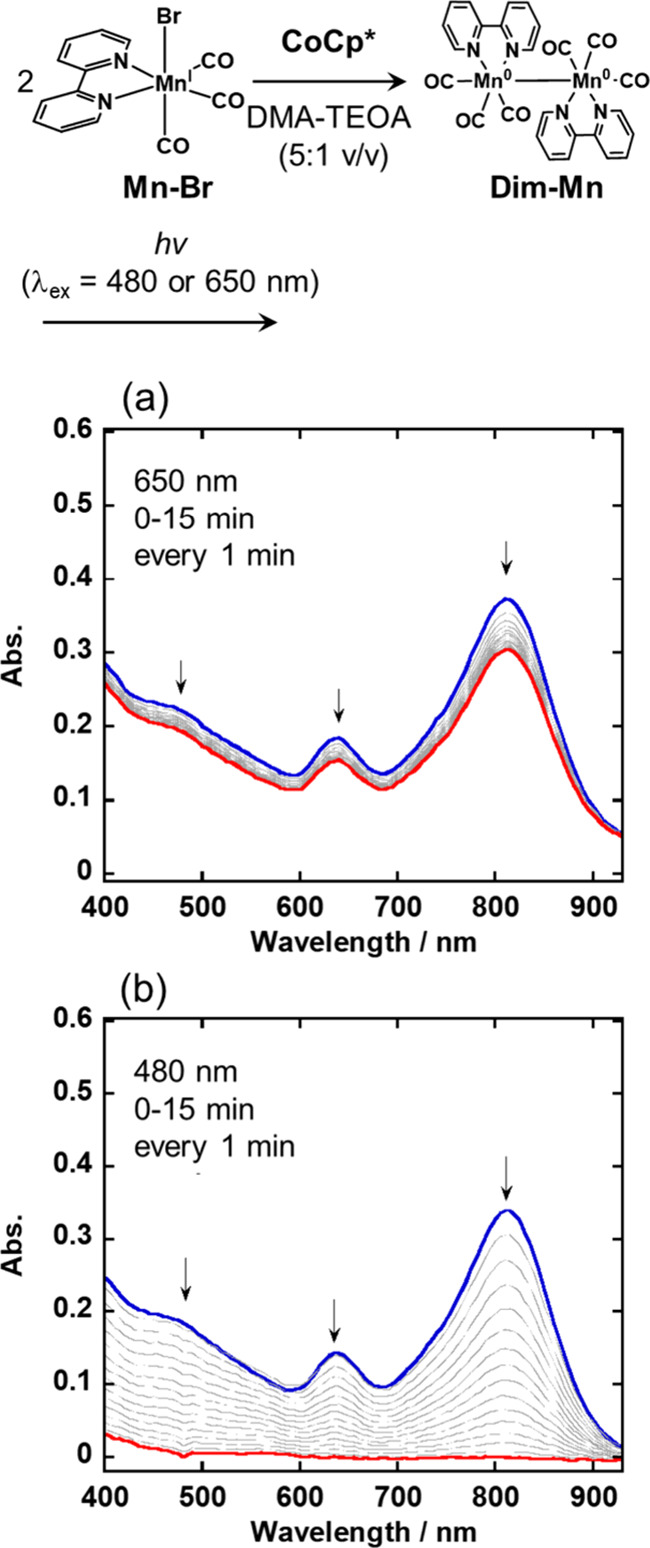
UV–vis absorption spectral changes of *in situ* synthesized **Dim-Mn** during irradiation in DMA-TEOA (5:1, v/v) under Ar atmosphere: (a) λ_ex_ = 650 nm and (b) λ_ex_ = 480 nm.

During irradiation at λ_ex_ = 650 nm (Figure 2a), the absorption attributed to **Dim-Mn** was slightly decreased (by 18% based on the spectrum before irradiation). After discontinuation of the irradiation, some parts of the reduced absorption bands of **Dim-Mn** (5% based on the spectrum before irradiation) reappeared within a few minutes (Figure S2a).

During irradiation at λ_ex_ = 480 nm (Figure 2b), on the other hand, the absorption intensity of the bands of **Dim-Mn** was observed to decrease drastically, and these absorption bands almost completely disappeared after irradiation for 15 min. In this experiment, **Dim-Mn** (4% based on the spectrum before irradiation) was recovered after the irradiation was discontinued (Figure S2b), after which CO was detected in the gas phase of the reactor. These results indicate that the photochemical reaction of **Dim-Mn** at λ_ex_ = 480 nm proceeds to decompose this dimer with the loss of the CO ligands, as elaborated in our previous report.^45^

**Figure 3:**
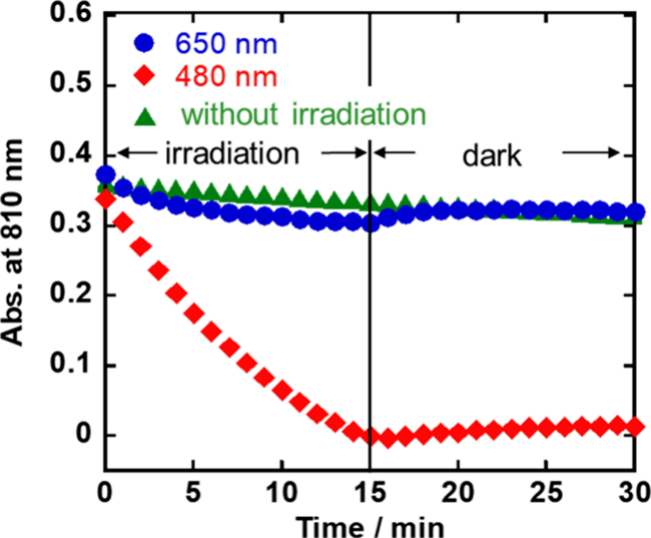
Time profile for absorbance changes of **Dim-Mn** at λ = 810 nm. Blue closed circles: irradiation at λ_ex_ = 650 nm, red rhombuses: irradiation at λ_ex_ = 480 nm, green triangles: without irradiation (dark condition). Irradiation was discontinued after 15 min.


Figure 3 shows the changes in the absorbance of **Dim-Mn** at λ = 810 nm as a function of time, with excitation at λ_ex_ = 650 nm (blue closed circles) and 480 nm (red rhombuses), along with the measurements made without irradiation (green triangles). It should be noted that, even in the “dark” condition, i.e., without irradiation using the irradiation apparatus, the absorption at λ = 810 nm decreased by 13% after the solution was maintained at room temperature for 30 min. This slow decomposition of **Dim-Mn** should be induced by the light source for measuring the *in situ* UV–vis absorption spectra. The amount whereby the absorption of **Dim-Mn** decreased after irradiation at λ_ex_ = 650 nm for 15 min, after which the solution was kept in the dark for 15 min (blue closed circles in Figure 3), was similar to (slightly less than) that when the solution was maintained without irradiation (green triangles). Moreover, irradiation of **Dim-Mn** at λ_ex_ = 650 nm produced chemical species, some of which led to the recovery of **Dim-Mn**. This strongly suggests that irradiation at λ_ex_ = 650 nm induces the homolytic cleavage of the Mn(0)–Mn(0) bond to produce the Mn(0) mononuclear complex, that is, [Mn^0^(bpy)(CO)_3_] (**Mn**^•^), and that the coupling reaction of two molecules of **Mn**^•^ induces the recovery of **Dim-Mn** (eq 1). As no obvious absorption attributable to **Mn**^•^ was detected in this experiment, the absorption of **Mn**^•^ in the visible region was expected to be relatively weak (Figure S3). In the photoreaction using 480 nm light, two photochemical reactions, namely, the loss of CO ligands and cleavage of the Mn(0)–Mn(0) bond, should proceed, with the former reaction being irreversible and probably dominant (eq 2). Hartl et al. also reported that the homolytic cleavage of **Dim-Mn** proceeded simultaneously with decomposition at λ_ex_ = 405 nm in their photoassisted electrochemical CO_2_ reduction system.^46^

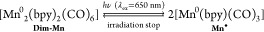
1
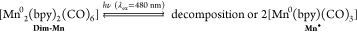
2It is noteworthy that the photochemical reactivities of **Dim-Mn** strongly depend on the wavelength of the irradiated light; namely, **Dim-Mn** does not follow Kasha’s rule, more obviously compared to [Mn^0^_2_(diimine)(CO)_8_] and [Mn^0^_2_(CO)_10_].^48−56^ In the photochemical reaction of **Dim-Mn** under irradiation with higher energy light (480 nm), irreversible decomposition involving the release of CO proceeds from a higher excited state or states. It competes with internal conversion to the lower excited state, which induces the homolytic cleavage of the Mn(0)–Mn(0) bond (Scheme S1).

**Figure 4:**
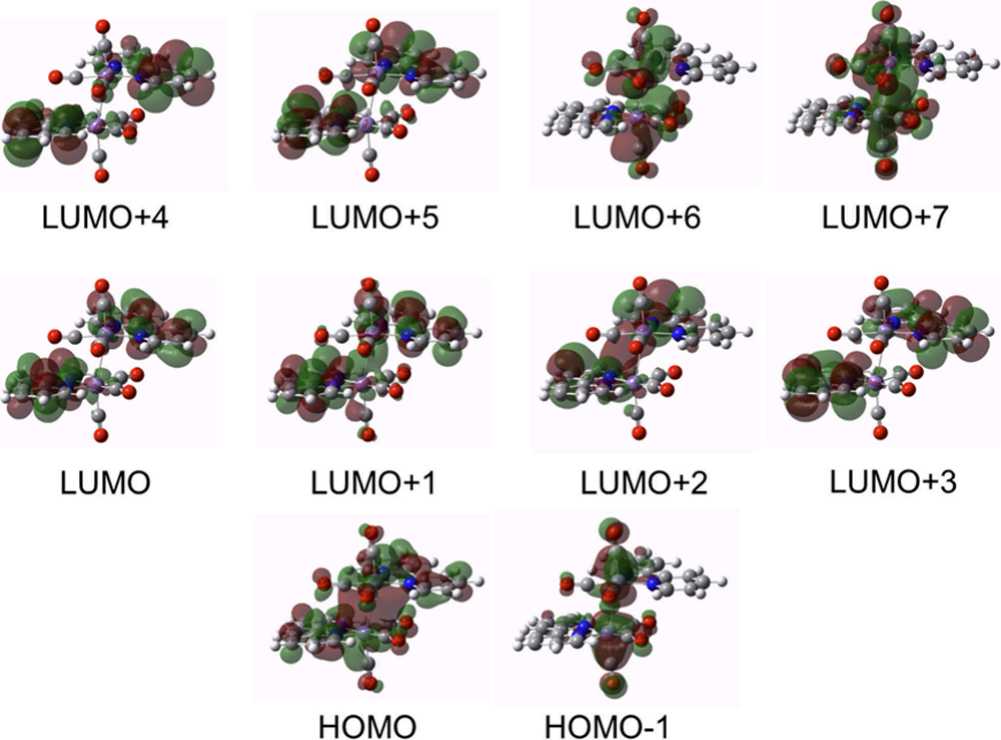
Molecular orbitals of **Dim-Mn**.

To clarify the dependence of the photochemical reactivity of **Dim-Mn** on the wavelength of the light, density functional theory (DFT) calculations were conducted as follows: The structure of **Dim-Mn** (Figure S4) was optimized by def2-SVP/PBE1PBE with the solvent effect of DMA,^59^ and the obtained structural parameters were quite similar to those of the reported crystal structure of **Dim-Mn**.^60^Figure 4 shows the molecular orbitals of HOMO, HOMO–1, and LUMO – LUMO+7, and the orbital distributions and energies of these molecular orbitals are summarized in Table S1. The HOMO is mainly distributed across the bonding σ orbital between Mn(0)–Mn(0) (54%). Conversely, HOMO–1 primarily consists of the bonding orbitals between the Mn(0) center and the carbonyl ligands (mainly π backbonding orbitals; the distributions of Mn and CO were 70 and 24%, respectively). LUMO and LUMO+2 – LUMO+5 are primarily composed of the π* orbital of bpy (92–96%), whereas LUMO+1 is composed not only of the π* orbitals of the bpy ligand (82%) but also of the σ* antibonding orbital between Mn(0)–Mn(0) (14%). LUMO+6 and LUMO+7 mainly consisted of antibonding orbitals between the Mn(0) center and the carbonyl ligands. The odd electrons in the higher excited states contributing to CO release are probably localized in HOMO–1, LUMO+6, and/or LUMO+7, which are constructed from the antibonding orbital between Mn(0) and the CO ligands, as described above. The homolytic cleavage of the Mn(0)–Mn(0) bond was related to the HOMO and/or LUMO+1.

**Figure 5:**
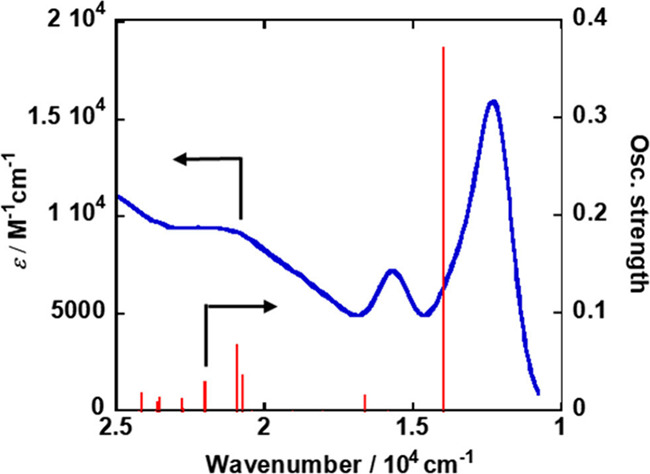
Oscillator strength of **Dim-Mn** from TD-DFT and UV–vis absorption spectrum of **Dim-Mn** measured in DMA-TEOA (5:1 v/v).

Figure 5 shows the absorption spectrum of **Dim-Mn** obtained from time-dependent (TD)-DFT calculations, along with the UV–vis absorption spectrum measured in DMA-TEOA (5:1 v/v). The shape and oscillator strength of each transition were in relatively good agreement with the UV–vis absorption spectra of **Dim-Mn**, even though the calculated values of the characteristic transitions at 13989 and 16649 cm^–1^ were higher than those of the corresponding absorption bands of the UV–vis spectrum at 12700 and 15625 cm^–1^.

The calculated oscillator strengths for each transition are summarized in Table 1. The absorption with the strongest oscillator strength at the lowest wavenumber (13989 cm^–1^) was assigned to the transition from the HOMO to the LUMO. The transitions from the HOMO to the LUMO+1, LUMO+2, and LUMO+3 contributed significantly to the oscillator strength at 16649, 20749, and 20937 cm^–1^, respectively. As described above, these transitions from the HOMO can weaken the Mn(0)–Mn(0) bond because the HOMO is mainly located on the σ bond of the Mn(0)–Mn(0) bond. This is in good agreement with the experimental results; that is, irradiation at λ_ex_ = 650 nm (15385 cm^–1^) induced only Mn(0)–Mn(0) bond cleavage. In contrast, the excitations contributed by the HOMO–1, LUMO+6, and LUMO+7, such as those at 22023, 22774, 23556, and 23620 cm^–1^ should weaken the Mn(0)-CO bonds, owing to the removal of an electron from the bonding orbital and/or injection of the electron to the antibonding orbitals. Thus, excitation with short-wavelength light, such as λ_ex_ = 480 nm (20833 cm^–1^), should cause the decomposition of **Dim-Mn**, which would involve the release of CO.

**Table 1:** Oscillator Strength of Each Transition up to 24000 cm^–1^ and Molecular Orbitals Involved in These Transitions, Based on the TD-DFT Calculations

Wavenumber/cm^–1^	Oscillator strength	Transition (Contribution ratio)^a^
13989	0.3711	HOMO→LUMO (100%)
16649	0.0152	HOMO→LUMO+1 (97%)
20749	0.036	HOMO→LUMO+2 (95%)
20937	0.0673	HOMO→LUMO+3 (95%)
22023	0.0286	HOMO–1→LUMO (11%), HOMO→LUMO+4 (71%), HOMO→LUMO+7 (14%)
22774	0.0009	HOMO–1→LUMO (73%), HOMO→LUMO+4 (18%)
22790	0.0117	HOMO→LUMO+5 (93%)
23556	0.0124	HOMO–1→LUMO+1 (48%), HOMO→LUMO+6 (22%)
23620	0.0086	HOMO–1→LUMO (10%), HOMO→LUMO+7 (70%)

a Each orbital distribution is the sum of two Mn centers, two bpy centers, and six CO atoms.

Based on these experimental results and theoretical studies, we conclude that irradiation of **Dim-Mn** at long wavelengths, such as λ_ex_ = 650 nm, excludes the decomposition of **Dim-Mn** via the release of CO to selectively produce **Mn**^•^. We applied the photochemical reactivity of **Dim-Mn** to construct a highly durable photocatalytic system, which incorporates an Mn(I) complex as the catalyst and uses only long-wavelength light for irradiation.

### Development of a Durable Photocatalytic CO_2_ Reduction System Using the Mn(I) Catalyst and Red Light

**Figure 6:**
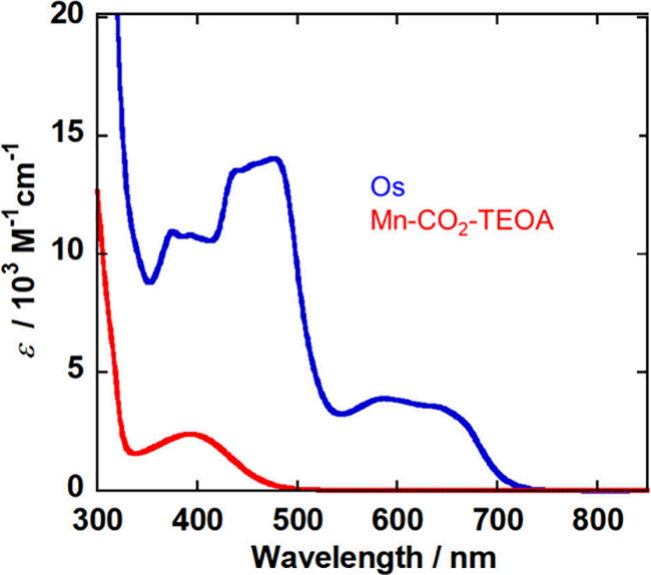
UV–vis absorption spectra of DMA-TEOA (5:1 v/v) solutions containing **Mn-CO**_**2**_**-TEOA** (red, under CO_2_ atmosphere) and **Os** (blue, under Ar atmosphere).

A photocatalytic CO_2_ reduction system that uses light with wavelengths longer than 600 nm was constructed by using the following system: *fac*-[Mn^I^(bpy)(CO)_3_(OC(O)OC_2_H_5_N(C_2_H_5_OH)_2_)] (**Mn-CO**_**2**_**-TEOA**) as the catalyst and [Os^II^(4,4′-dimethyl-2,2′-bipyridine)(5,5′-dimethyl-2,2′-bipyridine)_2_]^2+^ (**Os**) as the photosensitizer. The relatively strong absorption of this Os(II) trisdiimine complex at wavelengths longer than 600 nm (blue line in Figure 6) is attributed to direct excitation from the ground singlet state to the triplet excited state, which is partially allowed owing to the heavy-atom effect of the central Os(II). Its lowest ^3^MLCT (metal-to-ligand charge transfer) excited state have a lifetime of 46 ns in DMA-TEOA (5:1 v/v), which is sufficiently long to enable it to be used as a photosensitizer. This excited state of **Os** was reductively quenched by 1,3-dimethyl-2-phenyl-2,3-dihydro-1H-benzo[d]imidazole (**BIH**) as a sacrificial electron donor, as we previously reported.^61^**Mn-CO**_**2**_**-TEOA** does not absorb light at wavelengths longer than 480 nm (red line in Figure. 6).

**Figure 7:**
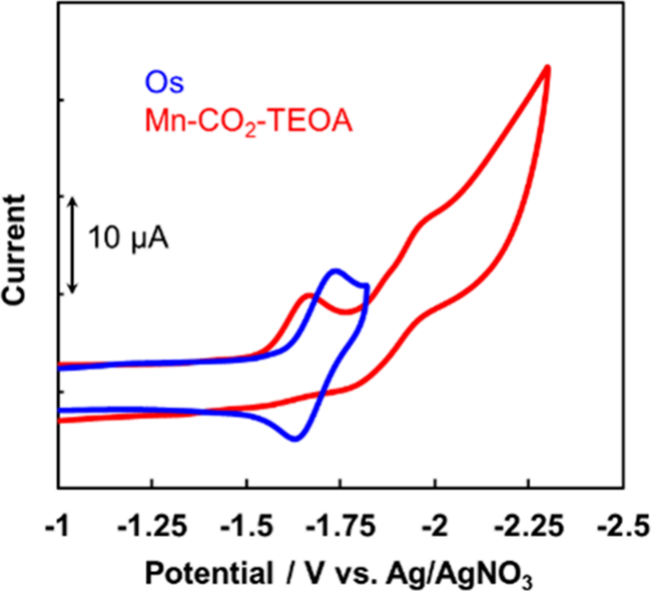
Cyclic voltammograms of **Mn-CO**_**2**_**-TEOA** (red, under CO_2_ atmosphere) and **Os** (blue, under Ar atmosphere): a DMA-TEOA (5:1 v/v) solution containing 0.5 mM of each complex and 0.1 M of Et_4_NBF_4_.

The cyclic voltammograms of **Mn-CO**_**2**_**-TEOA** and **Os** are shown in Figure 7. The one-electron reduced species of **Os** (**Os**^–^) (*E*_1/2_ = −1.77 V vs Fc/Fc^+^; the peak potential *E*_p_^red^ = −1.82 V) can reduce **Mn-CO**_**2**_**-TEOA** (*E*_p_^red^ = −1.79 V vs Fc/Fc^+^) whereas it cannot reduce **Dim-Mn** (*E*_p_^red^ = −1.89 V vs Fc/Fc^+^). This differs markedly from the reported photocatalytic CO_2_ reduction systems that rely on Cu(I)-complex photosensitizers in which the one-electron reduced species have more negative reduction potentials (−2.05 V vs Ag/AgCl and −2.01 V vs Fc/Fc^+^) than that of **Dim-Mn**, but these Cu(I) complexes can absorb only much shorter wavelength light (≤500 nm) than **Os**.^62,63^

**Figure 8:**
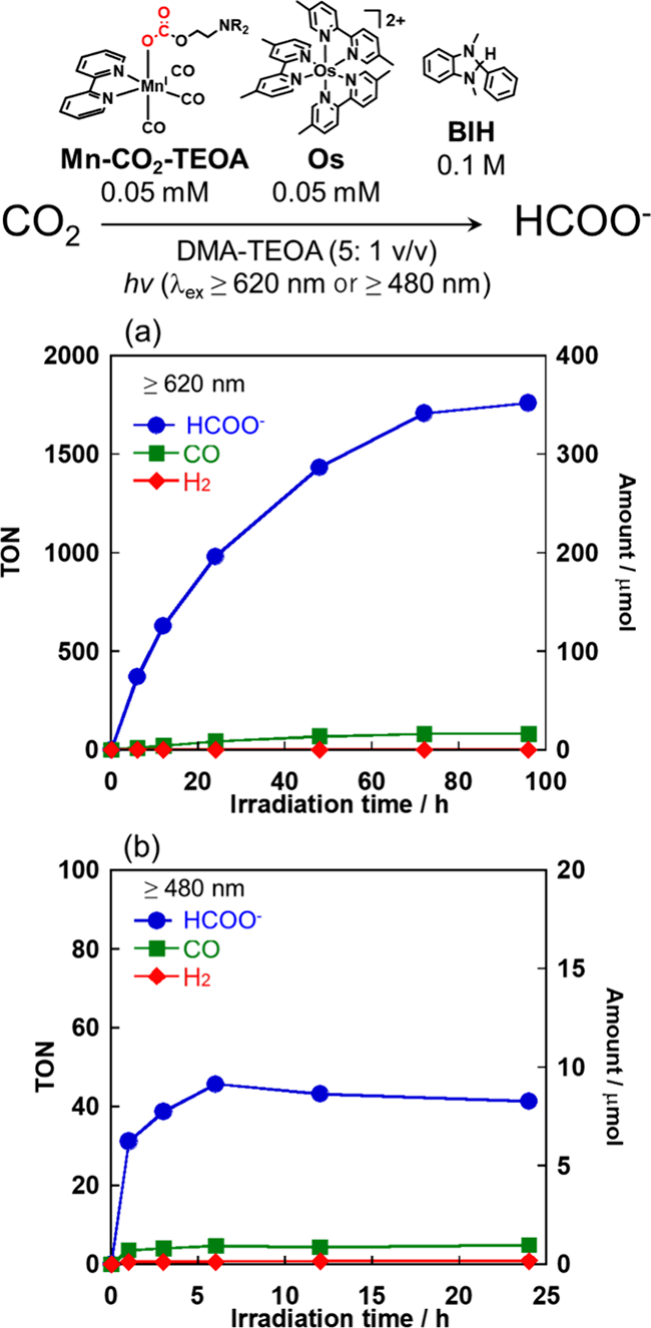
Time courses of the reaction products (HCOO^–^, CO, H_2_). Photocatalytic reactions were conducted in a DMA-TEOA (5:1 v/v) solution containing **Mn-CO**_**2**_**-TEOA** (0.05 mM), **Os** (0.05 mM), and **BIH** (0.1 M) under a CO_2_ atmosphere with irradiation at (a) λ_ex_ ≥ 620 nm and (b) λ_ex_ ≥ 480 nm.

As a typical photocatalytic CO_2_ reduction reaction, a CO_2_-saturated DMA-TEOA (5:1 v/v) mixed solution containing **Mn-CO**_**2**_**-TEOA** (0.05 mM), **Os** (0.05 mM), and **BIH** (0.1 M) was irradiated at λ_ex_ ≥ 620 nm (Figure 8a) or λ_ex_ ≥ 480 nm (Figure 8b) using a 500 W halogen lamp with appropriate cutoff filters. Under both photocatalytic reaction conditions, HCOO^–^ was produced with high selectivity (>95% at λ_ex_ ≥ 620 nm; 87% at λ_ex_ ≥ 480 nm). However, the durability of the photocatalytic system strongly depends on the wavelength of the irradiated light. Under irradiation at λ_ex_ ≥ 620 nm, HCOO^–^ was produced for up to 96 h, and the turnover number of HCOO^–^ (TON_HCOO–_) reached 1762 (352 μmol, selectivity >95%). CO (16 μmol, 4%) and H_2_ (0.2 μmol, < 1%) were detected as minor products. This selectivity is much higher than the reported systems using the similar Mn(I)-complex catalysts coupled with a Ru(II) photosensitizer (89%) or a Cu(II) dinuclear photosensitizer (74%).^45,63^ The total amount of reduction products (HCOO^–^, CO, H_2_) was 369 μmol, which was close to the amount of **BIH** used (400 μmol) because one molecule of **BIH** can supply two electrons in the photocatalytic reactions.^64^ The quantum yield of HCOO^–^ formation in the photocatalytic reaction using 650 nm monochromic light (1.3 × 10^–7^ einstein/s) was 4.3%. At λ_ex_ ≥ 480 nm, conversely, the formation of HCOO^–^ ceased after 6 h, and TON_HCOO–_ was only 41. Increasing the reaction time to 24 or 48 h did not increase the amount of HCOO^–^ that formed.

We conducted a photocatalytic reaction using ^13^CO_2_ to determine the carbon source of the produced HCOO^–^. A DMA-TEOA (5:1 v/v) solution containing 1 mM of **Mn-CO**_**2**_**-TEOA** and **Os** and 0.1 M of **BIH** was irradiated under a ^13^CO_2_ atmosphere at λ_ex_ ≥ 620 nm for 19 h. Under this reaction condition, almost only H^13^COO^–^ was detected by GC-MS (Figure S5). A control experiment under an Ar atmosphere, i.e., without CO_2_, only trace amounts of H_2_ and HCOO^–^ were detected. These results clearly show HCOO^–^ was derived from the reduction of CO_2_. In addition, as another control experiment, irradiation to the solution in the absence of the Mn catalyst did not catalytically produce HCOO^–^ and CO. Therefore, **Os** and their decomposition products during the photocatalytic reaction did not work as a catalyst for CO_2_ reduction, and the Mn complex worked as the catalyst for CO_2_ reduction.

**Figure 9:**
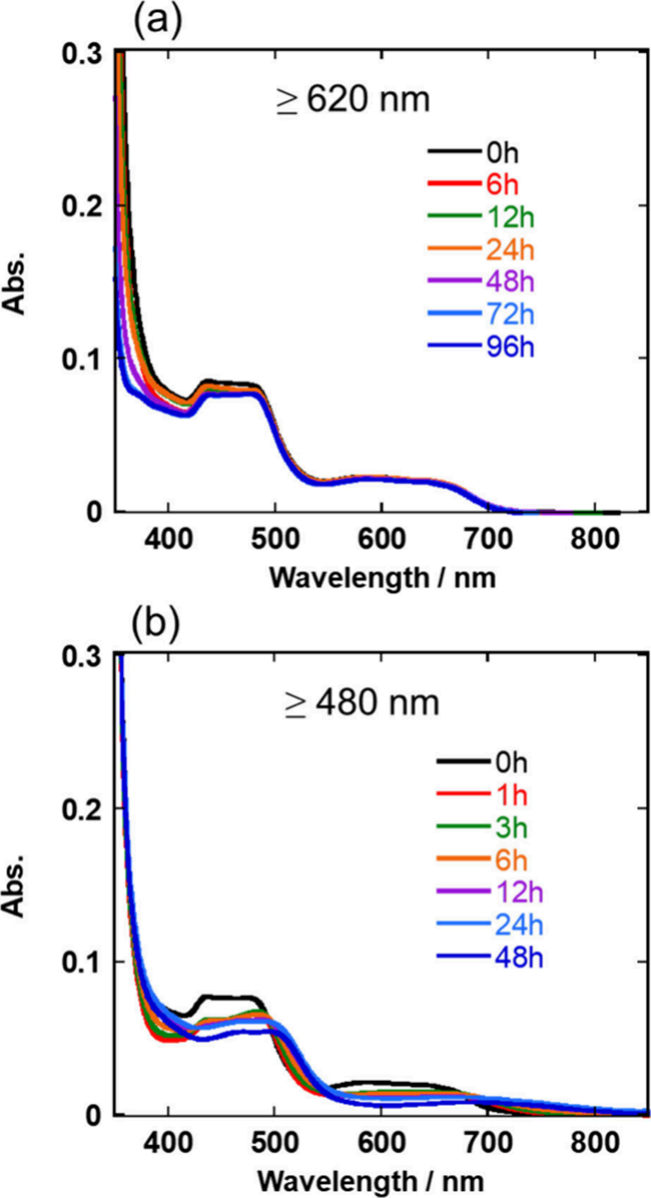
UV–vis absorption spectra recorded after the photocatalytic reactions. These reactions were conducted in a DMA-TEOA (5:1 v/v) solution containing **Mn-CO**_**2**_**-TEOA** (0.05 mM), **Os** (0.05 mM), and **BIH** (0.1 M) under a CO_2_ atmosphere with irradiation at (a) λ_ex_ ≥ 620 nm and (b) λ_ex_ ≥ 480 nm. The solutions were exposed to air when these spectra were recorded.

Figure 9a shows the UV–vis absorption spectra recorded after the photocatalytic reactions; the characteristic ^3^MLCT absorption band of **Os** (in the range 550–700 nm) did not change in the solutions irradiated at λ_ex_ ≥ 620 nm. Under irradiation at λ_ex_ ≥ 480 nm, meanwhile, the UV–vis absorption spectra changed drastically even after irradiation for 1 h (Figure 9b). These results clearly indicate that although **Os** decomposed within this time scale during the photocatalytic reaction using shorter-wavelength light, such decomposition did not occur in the photocatalytic reaction using light of λ_ex_ ≥ 620 nm.

**Figure 10:**
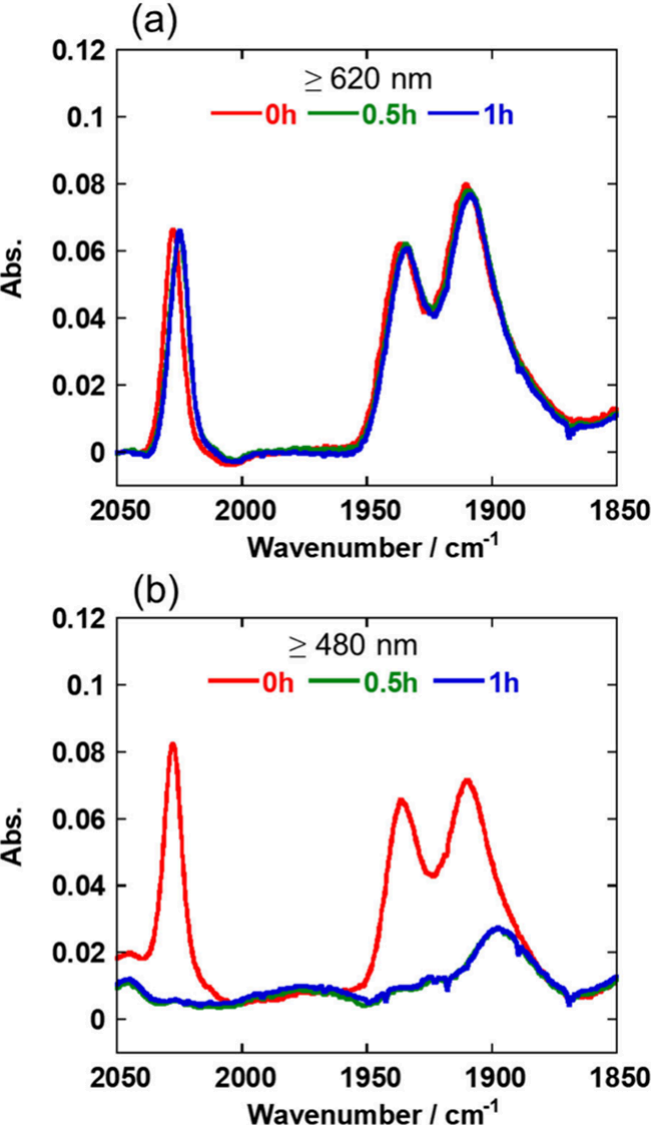
IR spectra measured before (red) and after photocatalytic reaction (green and blue). Photocatalytic reactions were conducted in a DMA-TEOA (5:1 v/v) solution containing **Mn-CO**_**2**_**-TEOA** (0.5 mM), **Os** (0.05 mM), and **BIH** (0.1 M) under a CO_2_ atmosphere with irradiation at (a) λ_ex_ ≥ 620 nm and (b) λ_ex_ ≥ 480 nm. The reacted solutions were exposed to air when these spectra were measured.

The structural changes in the Mn(I) catalyst were examined by analyzing the FT-IR spectra of the reaction solutions before and after the photocatalytic reactions. In these experiments, a higher concentration of the Mn catalyst (0.5 mM) was used to ensure sufficient sensitivity for the FT-IR measurements. Irradiation for 30 min at λ_ex_ ≥ 480 nm resulted in the disappearance of the stretching bands of the CO ligands of **Mn-CO**_**2**_**-TEOA** (ν_CO_ = 2028, 1936, 1910 cm^–1^). New absorptions attributable to the formation of other carbonyl complexes were not observed (Figure 10b). Conversely, irradiation at λ_ex_ ≥ 620 nm had no effect on the tricarbonyl structure of the Mn(I) catalyst, which remained entirely intact even after irradiation for 1 h (Figure 10a: The slight difference in the spectra before and after the photocatalytic reaction is disction Mechanism of the photocatalytic reaction). This result indicates that the Mn(I) catalyst does not decompose (or decomposes very slowly) via the release of the CO ligands under irradiation at λ_ex_ ≥ 620 nm. Thus, the durability of the photocatalytic system based on the Mn(I) catalyst was improved by selecting the wavelength for irradiation as λ_ex_ ≥ 620 nm to prevent the decomposition of the Mn catalyst; meanwhile, irradiation at λ_ex_ ≥ 480 nm induces the decomposition of the Mn catalyst, thereby inhibiting the photocatalysis of this system. The decomposition of the Os(II) photosensitizer during the photocatalytic reaction probably proceeded via the photoexcitation of its one-electron-reduced species **Os**^–^, which is likely to have accumulated in the reaction solution after the Mn catalyst had decomposed.

At the end of this section on the photocatalysis of the system consisting of the Mn catalyst, it might be noteworthy to compare this and the photocatalysis of the system using the Re(I)-complex catalyst instead of the Mn catalyst in the similar reaction condition. We already reported the photocatalytic CO_2_ reduction system consisting of [Re(dmb){P(*p*-Cl-phenyl)_3_}_2_(CO)_2_}]^+^ (dmb = 4,4′-dimethyl-bpy), **Os**, and BIH, which mainly produced CO and TON_CO_ was 364 after irradiation for 20 h.^61^ Since the reaction condition between the Re and Mn systems are similar except of the catalyst itself and TON_HCOOO–_ was 984 after 24 h irradiation, we can say that the system using **Mn-CO**_**2**_**-TEOA**, **Os**, and **BIH** is a very efficient and durable photocatalytic system.

### Mechanism of the Photocatalytic Reaction

**Figure 11:**
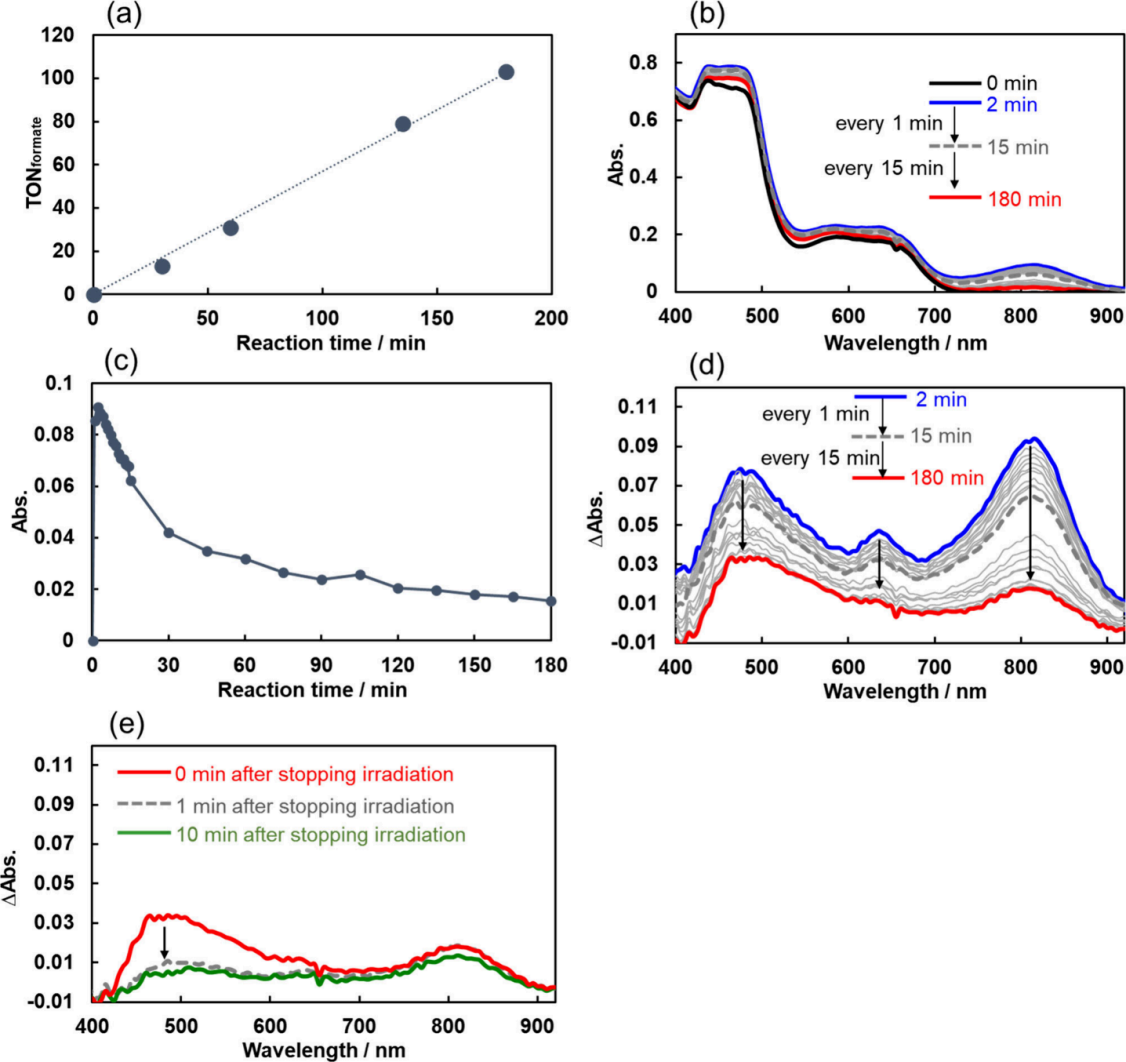
(a) Time dependence of HCOO^–^ formation. (b) Spectral changes in the UV–vis absorption during the photocatalytic reaction; a DMA-TEOA (5:1 v/v) solution containing **Mn-CO**_**2**_**-TEOA** (0.05 mM), **Os** (0.05 mM), and **BIH** (0.1 M) was irradiated at λ_ex_ = 650 nm under a CO_2_ atmosphere. These UV–vis spectra in the periods 0–14 min and 15–180 min were measured separately due to the different measurement intervals. (c) Time profile for the absorbance change at 810 nm during the photocatalytic reaction. (d) UV–vis differential spectra (relative to the spectrum recorded before irradiation), which were obtained from the UV–vis absorption spectra shown in (b). (e) UV–vis differential spectra immediately after the photocatalytic reaction for 180 min (red; same as the red line in (d)), and after this solution was kept in the dark after irradiation for 1 min (gray dashed) and 10 min (green).

The photocatalytic reaction begins with the photoinduced reduction of **Os**. The ^3^MLCT-excited **Os** was quenched by **BIH**, resulting in the formation of **Os**^**–**^ (Figure S6) with the quenching rate constant of *k*_q_ = 1.8 × 10^8^ M^–1^ s^–1^.^61^ This should be reductive quenching because of its exergonicity. **Os**^**–**^ should transfer one electron to **Mn-CO**_**2**_**-TEOA** because of its exergonicity as well to produce the one-electron-reduced species ([**Mn-CO**_**2**_**-TEOA**]^−^). The reaction pathway following the formation of [**Mn-CO**_**2**_**-TEOA**]^−^ during the photocatalytic reaction was investigated by *in situ* UV–vis spectroscopy. Figure 11a shows time dependence of TON_HCOO–_ in the photocatalytic reaction with *in situ* UV–vis spectroscopy under a CO_2_ atmosphere and irradiation at λ_ex_ = 650 nm. The UV–vis spectra that were recorded at specific points in time during the photocatalytic reaction are shown in Figure 11b. In this photocatalytic reaction, HCOO^–^ continuously formed during 180 min of irradiation (Figure 11a).

However, even after 2 min of irradiation, the absorption spectrum had changed significantly. The broad absorptions at λ_max_ = 650 and 810 nm were attributed to **Dim-Mn** owing to the similarity of the spectra (Figure 5, Figure S1). This indicates that detachment of the carbonate ester ligand ((HOC_2_H_4_)_2_NC_2_H_4_OC(O)O^–^) from [**Mn-CO**_**2**_**-TEOA**]^–^ proceeded to generate **Mn**^•^, which underwent dimerization to form **Dim-Mn**. Figure 11c shows the absorption changes at 815 nm, which can be attributed to the absorption of **Dim-Mn**, during the photocatalytic reaction. After irradiation for 2 min, the concentration of the accumulated **Dim-Mn** reached a maximum of approximately 0.006 mM (24% of the initial concentration of **Mn-CO**_**2**_**-TEOA**). Thereafter, the absorption of **Dim-Mn** gradually decreased, but slowed down after irradiation for 30 min, suggesting that a “quasi”-photostationary state had been reached after 30 min. The continuous slow decrease should be mainly induced by the light source of the spectrometer that was used to measure the *in situ* UV–vis absorption spectra (D_2_/I_2_ mixed lamp). After irradiation for 180 min at λ_ex_ = 650 nm, TON_HCOO–_ exceeded 100, and approximately 25% of the maximum accumulated amount (0.006 mM) of **Dim-Mn** remained.

The differential spectra at λ ≤ 600 nm in Figure 11d, acquired by irradiating the solution for 2 min, differed from that of the synthesized **Dim-Mn** (Figure S1a). Further continuous irradiation induced a larger difference between observed spectra and the spectra of **Dim-Mn** (for example, the red line in Figure 11d). This suggests that other Mn complex(es) had formed in addition to **Dim-Mn** during the photocatalytic reaction. The differential spectral changes in the solution at 180 min of irradiation and after discontinuation of the irradiation are displayed in Figure 11e. The absorption at λ ≤ 650 nm decreased when the solution was kept for 1 min after the irradiation was discontinued (gray dashed line in Figure 11e). In contrast, the absorption at wavelengths longer than approximately 650 nm, the characteristic absorption of **Dim-Mn**, remained largely unchanged even after keeping the solution in the dark for 10 min (green line in Figure 11e).

**Figure 12:**
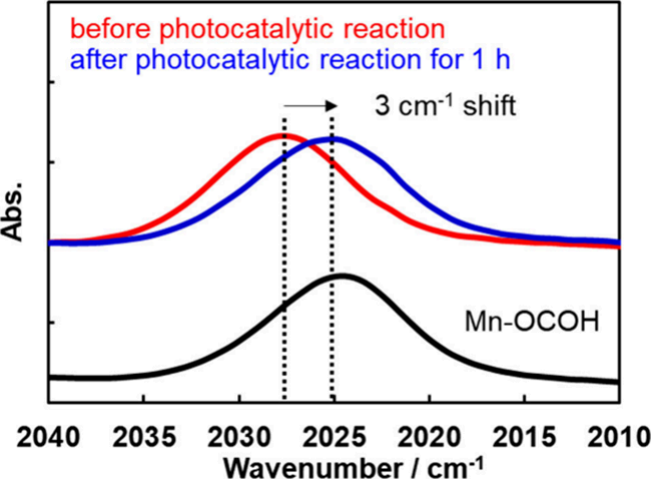
Comparison of FT-IR spectra recorded before/after the photocatalytic reaction displayed with the spectrum of **Mn-OCOH**. The spectra acquired before/after the photocatalytic reaction shown here are enlargements of the 2040–2010 cm^–1^ spectral region in Figure 10a. The spectrum of **Mn-OCOH** was measured in DMA-TEOA (5:1 v/v).

The changes in the FT-IR spectra before and after the photocatalytic reaction (shown in Figure 10a) were analyzed in detail: Figure 12 shows an enlargement of the FT-IR absorption attributed to the total-symmetrical stretching vibration of the CO ligands of the Mn complex before (red) and after 1 h of the photocatalytic reaction (blue). The ν_CO_ was red-shifted to a slightly lower energy (2028 → 2025 cm^–1^). Likewise, the ν_CO_s of the other two bands also shifted to lower energies from ν_CO_ of 1936 and 1910 cm^–1^ to ν_CO_ of 1934 and 1909 cm^–1^, respectively (Figure S7). The IR absorptions observed after the photocatalytic reaction were very similar to those of the Mn(I) formate complex, *fac*-[Mn^I^(bpy)(CO)_3_{OC(O)H}] (**Mn-OCOH**) (black line in Figure 12; 2025, 1935, and 1909 cm^–1^). Notably, no spectral changes were observed when Ar was bubbled through the reacted solution after the photocatalytic reaction had proceeded for 1 h (Figure S8). This indicates that the starting complex, i.e., **Mn-CO**_**2**_**-TEOA** did not remain in the solution (at least as a major component) after the photocatalytic reaction for 1 h because **Mn-CO**_**2**_**-TEOA** (ν_CO_ = 2028, 1936, 1910 cm^–1^) was converted to a mixture of *fac*-[Mn^I^(bpy)(CO)_3_(DMA)]^+^ (ν_CO_ = 2039, 1944, 1933 cm^–1^) as the major species and *fac*-[Mn^I^(bpy)(CO)_3_(OC_2_H_4_N(C_2_H_4_OH)_2_] (ν_CO_ = 2017, 1900 cm^–1^) as the minor species in the DMF-TEOA solution (eq 3) after a solution containing **Mn-CO**_**2**_**-TEOA** was bubbled with Ar.^59^
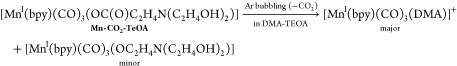
3

**Figure 13:**
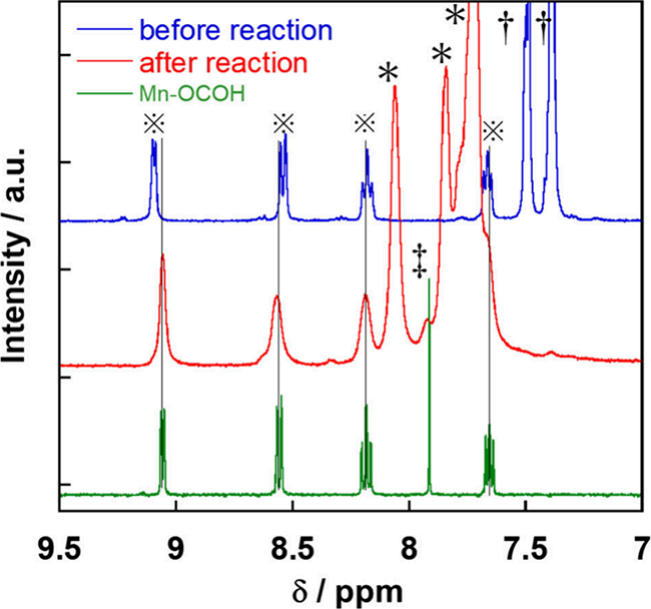
^1^H NMR spectra recorded before/after the photocatalytic reaction with the spectrum of **Mn-OCOH** in DMSO-*d*_6_. DMSO-*d*_6_-TEOA (5:1 v/v) containing 30 mM of **Mn-CO**_**2**_**-TEOA**, 0.39 mM of **Os**, and 0.1 M of **BIH** was irradiated for 60 h at λ_ex_ ≥ 620 nm under CO_2_ atmosphere. × with dots: bpy ligand of Mn complexes, ‡: formate ligand,†: BIH, *: BI^+^.

In the ^1^H NMR spectrum measured after the photocatalytic reaction in DMSO-*d*_6_-TEOA for 60 h, the signals attributed to the bpy ligand were observed at δ = 9.09, 8.54, 8.18, and 7.66 ppm and the signal of the coordinated formate (−OC(O)-***H***) was detected at δ = 7.92 ppm (red spectrum in Figure 13). Although the peaks were observed to have broadened, probably due to the formation of paramagnetic Mn species as minor products,^45^ the chemical shifts were in good agreement with those of the ^1^H NMR spectrum of **Mn-OCOH** (Figure 13). This also strongly suggests that certain parts of **Mn-CO**_**2**_**-TEOA** were converted to the formate complex **Mn-OCOH** during the photocatalytic reaction over 1 h. This structural change of **Mn-CO**_**2**_**-TEOA** during the photocatalytic reaction was followed by quasi-*in situ* FT-IR measurement as well: the photocatalytic reaction solution in an IR cell was irradiated at λ_ex_ ≥ 640 nm, and FT-IR spectra were measured within 10 s after the irradiation was discontinued (Figure S9). The spectra indicated that the conversion of **Mn-CO**_**2**_**-TEOA** to **Mn-OCOH** commenced even during the initial stage of the photocatalytic reaction, within the first 3 min of irradiation, and that this structural change was completed within 30 min of irradiation. Thus, most of the **Mn-CO**_**2**_**-TEOA** should be converted into **Dim-Mn** and **Mn-OCOH** during the initial stage of the photocatalytic reaction (eq 4), and these two products should function as the main catalysts for CO_2_ reduction to produce HCOO^–^. Noteworthy is that considering the similarity between the UV–vis absorption spectra of **Mn-OCOH** and **Mn-CO**_**2**_**-TEOA** (Figure S10), together with the absence of any characteristic absorption at λ ≥ 480 nm, the formation of **Mn-OCOH** would not be expected to significantly affect the UV–vis absorption spectra during the photocatalytic reaction (Figure 11).
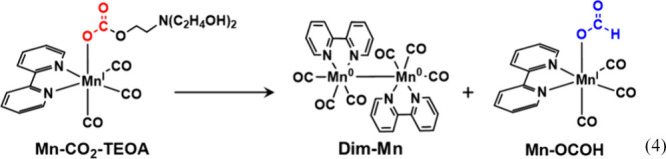
4

**Dim-Mn** is the one-electron-reduced product resulting from the dimerization of **Mn**^•^, whereas **Mn-OCOH** is formed as a result of the two-electron reduction of CO_2_ combined with protonation in the complex. These two complexes coexist in the reaction solution during the photocatalytic reaction. However, the *in situ* UV–vis differential spectra shown in Figure 11d and 11e cannot be explained on the basis of these two Mn species alone. Thus, another intermediate should form between **Dim-Mn** and **Mn-OCOH**. It was reported that hydride complexes with various metals react with CO_2_ to be converted to the formate complexes^65^ via insertion of CO_2_ into the metal hydride bond (eq 5). Kang et al. and Reisner et al. experimentally observed the formation of the Mn(I) hydride species *fac*-[Mn^I^(diimine)(CO)_3_H] in a heterogeneous photocatalytic system involving a Mn(I) diimine tricarbonyl complex immobilized on a solid surface.^66,67^
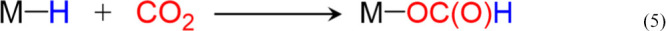
5

**Figure 14:**
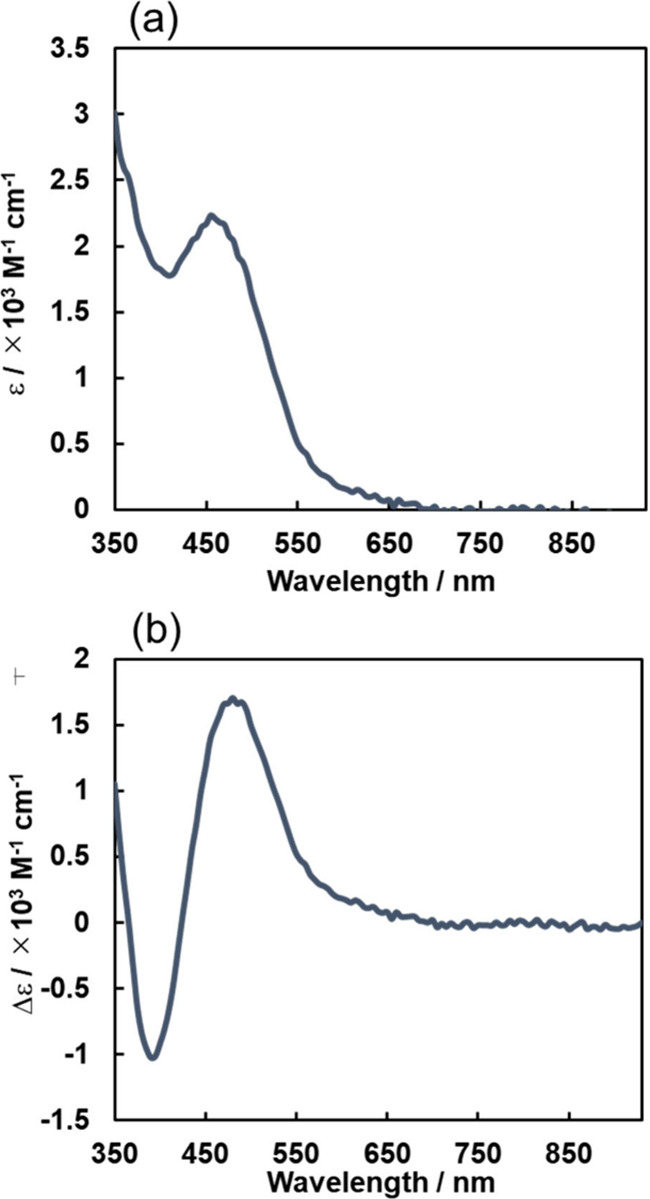
(a) UV–vis absorption spectra of *in situ* synthesized **Mn-H** in DMA-TEOA (5:1 v/v). (b) Differential spectrum obtained by subtraction of the spectrum of **Mn-CO**_**2**_**-TEOA** as the reference from that of **Mn-H**.

To verify the formation of *fac*-[Mn^I^(bpy)(CO)_3_H] (**Mn-H**) as a candidate intermediate during the photocatalytic reaction, we conducted the following experiments. A solution containing **Mn-H** was prepared by the chemical reduction of **Mn-Br** to the two-electron-reduced species [Mn(bpy)(CO)_3_]^−^ (**TERS-Mn**) using CoCp* as the reductant, and the subsequent protonation of **TERS-Mn** with NH_4_PF_6_ as the proton source in a glovebox filled with Ar (eq 6). The formation of **Mn-H** as the main product was confirmed by FT-IR spectroscopy: the ν_CO_s of the produced **Mn-H** (1986 and 1885 cm^–1^, shown in Figure S11) were very similar to the reported values (1989 and 1892 cm^–1^ in THF).^68^ This spectroscopy also indicated the formation of **Dim-Mn** as a minor product. The UV–vis absorption spectrum of **Mn-H** was obtained by subtracting the spectra of residual **Dim-Mn** and [CoCp*]^+^ from the recorded spectrum (details are shown in Figure S12 and the Experimental section). The corresponding spectra are shown in Figure 14a. As a reference spectrum for the following experiments, the UV–vis absorption spectrum of **Mn-CO**_**2**_**-TEOA** as the starting complex (Figure 6) was subtracted from that of **Mn-H** (Figure 14a) to give the differential spectrum shown in Figure 14b, which showed a characteristic absorption peak at λ_max_ = 480 nm and no strong absorption at λ ≥ 650 nm.
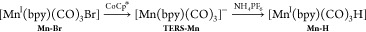
6

As described above, the *in situ* UV–vis differential absorption spectra before and after the photocatalytic reaction (Figure 11d) revealed persistent absorption at λ_max_ ∼ 480 nm during the photocatalytic reaction. This absorption did not originate from either **Dim-Mn** or **Mn-OCOH** (the UV–vis absorption spectrum of **Mn-OCOH** was very similar to that of the starting Mn(I) complex **Mn-CO**_**2**_**-TEOA**, as shown in Figure S9). Furthermore, most of this absorption at λ_max_ ∼ 480 nm diminished in the dark within 1 min after the photocatalytic reaction (Figure 11e). These results indicate that **Mn-H** was produced and accumulated in the reaction solution during the photocatalytic reaction and that it reacted relatively slowly with CO_2_.

**Figure 15:**
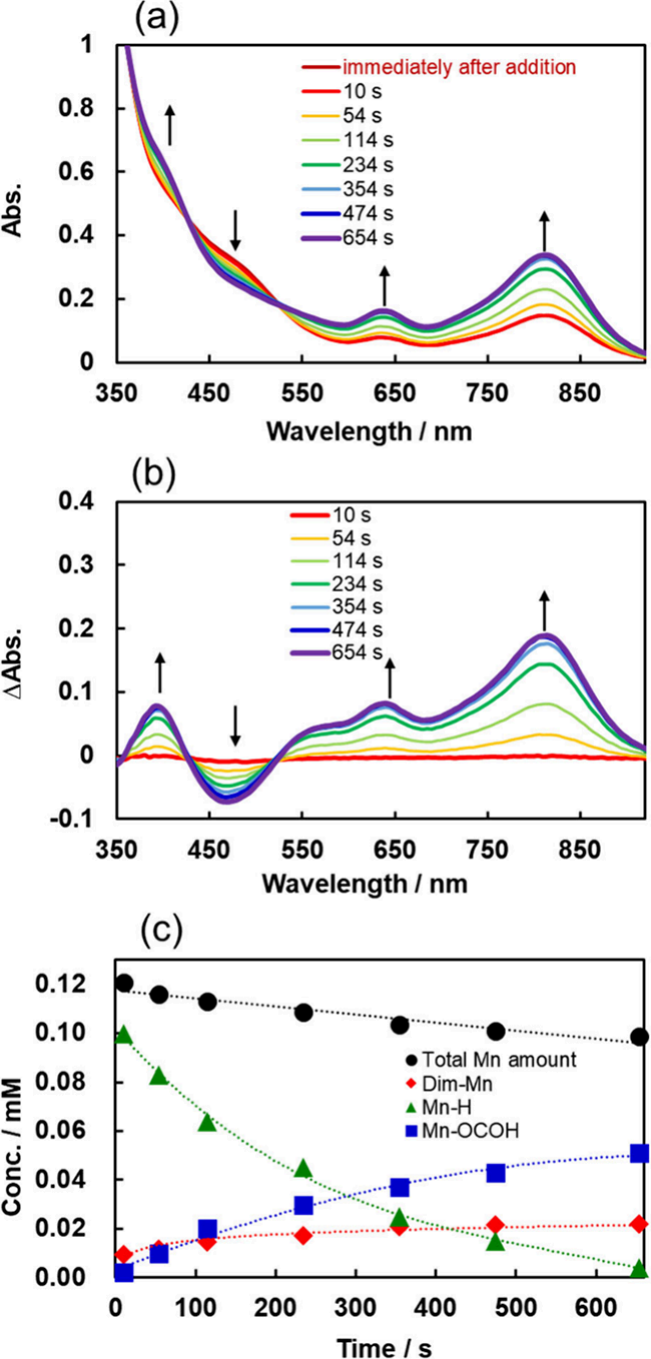
(a) UV–vis absorption spectral changes after mixing solutions containing **Mn-H** or saturated with CO_2_. (b) Differential spectra which were obtained by subtracting “immediately after addition” from the other observed spectra. (c) Concentration changes of the Mn complexes derived by fitting the observed *in situ* UV–vis absorption spectra during the reaction. The total Mn concentration (black solid circles) was 2 × [**Dim-Mn**] + [**Mn-H**] + [**Mn-OCOH**]. 0.2 mL of a DMA solution saturated with CO_2_ was added to a DMA-TEOA (5:1 v/v) solution containing approximately 0.11 mM of **Mn-H** in the dark.

The reactivity of **Mn-H** with CO_2_ in the dark was followed by using *in situ* UV–vis spectroscopy. A solution containing **Mn-H** was prepared by the aforementioned method (approximately 0.11 mM of **Mn-H**, 4.0 mL). Subsequently, 0.2 mL of a DMA solution saturated with CO_2_ was added to this solution, leading to the spectral changes, as shown in Figure 15a. Figure 15b shows the differential spectra which were obtained by subtracting “immediately after addition” (deep red line in Figure 15a) from the spectra recorded at various times after the mixing. The spectra continued to change for over 600 s. The absorption at λ = 480 nm, attributed to **Mn-H**, decreased, whereas the absorptions at λ_max_ = 635 and 810 nm, attributed to **Dim-Mn**, as well as at λ ∼ 400 nm, attributed to **Mn-OCOH**, increased. The spectra observed during the reaction of **Mn-H** and CO_2_ were fitted to the sum of the spectra of **Mn-H**, **Mn-OCOH**, **Dim-Mn**, and [CoCp*]^**+**^ (Figure S13), and the concentration changes of these Mn species were obtained by the fitting analysis (Figure 15c). This result indicates that the formation of **Mn-OCOH** was coupled with the decrease in **Mn-H**. The formation of **Mn-OCOH** proceeded via the insertion of CO_2_ into the Mn–hydride bond of **Mn-H** (eq 7). The conversion of **Mn-H** to **Dim-Mn** was also observed because the bimolecular reaction of **Mn-H** produces H_2_ and **Dim-Mn** (eq 8). This is in agreement with the reported finding that **Mn-H** was converted to **Dim-Mn** in the crystal phase.^68^ The gradual decrease in the total amount of Mn complexes during this reaction is probably explained by the induced decomposition of **Dim-Mn** by light irradiation, which is necessary for the measurement of the *in situ* UV–vis absorption spectra.
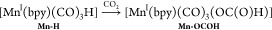
7
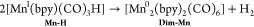
8

These results strongly indicate that the accumulation of **Mn-H** as another intermediate in the photocatalytic reaction solution should be induced by the relatively slow formation of **Mn-OCOH** during the photocatalytic reaction.

The spectroscopic results acquired during the photocatalytic reaction are summarized as follows. Electrochemical data of related compound are summarized in Table S2.

**Scheme 2:**
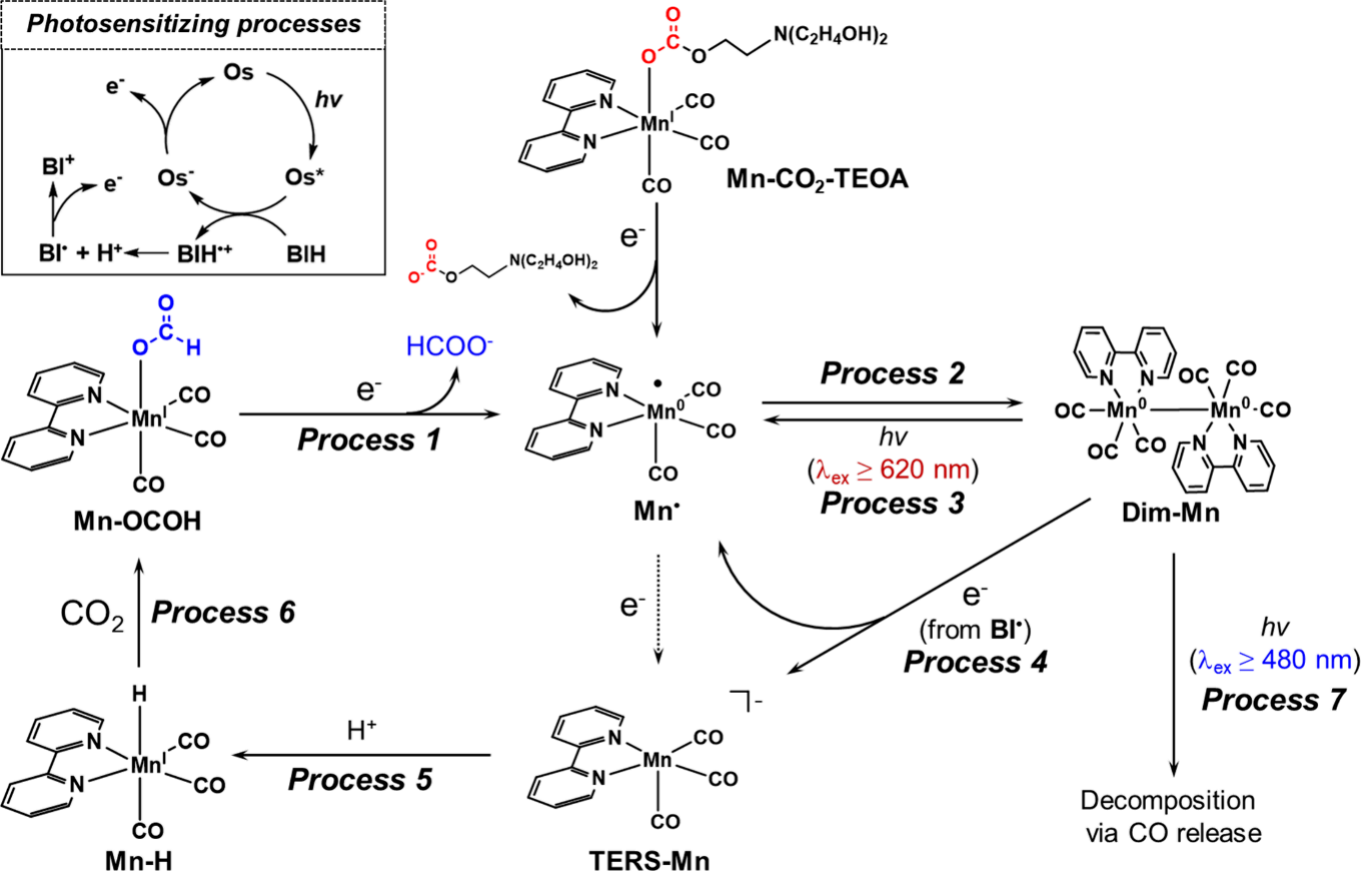
Reaction Mechanism of the Photocatalytic Reduction of CO_2_

(1) **Os**-Photosensitized electron transfer from **BIH** to **Mn-CO**_**2**_**-TEOA** starts the photocatalytic reaction. Reportedly, **BIH**^•+^ rapidly releases a proton to form **BI**^•^,^64^ which, with its high reduction potential (*E*_p_^ox^ = −2.06 V vs Fc/Fc^+^), can exergonically pass one electron to **Os**, **Mn-CO**_**2**_**-TEOA, Dim-Mn**, or **Mn•**.
(2) **Dim-Mn** can accept an electron from only **BI**^•^ owing to its high reduction potential (*E*_p_^red^ = −1.89 V vs Fc/Fc^+^) and is subsequently protonated to form **Mn-H**. **Mn-H** is converted to **Mn-OCOH** by slowly reacting with CO_2_. The main proton source should be **BIH**^•+^ that produced the photosensitizing process (Scheme 2).
(3) During the photocatalytic reaction, except for the initial stage, a “photostationary state” is established among **Dim-Mn**, **Mn-H**, and **Mn-OCOH**.

Based on these results, we can outline the photocatalytic cycle for producing HCOO^–^, as shown in Scheme 2. The electrons for the reduction of CO_2_ are supplied by **Os**^–^ and **BI**^•^, both of which are produced via the photosensitization of **Os** (enclosed within the square box in Scheme 2). Reduction of **Mn-CO**_**2**_**-TEOA** induces elimination of the carbonate ester ligand to give the 17-reduced species, and subsequently, **Mn**^•^ dimerizes to form **Dim-Mn** (Process 2). Upon excitation at λ_ex_ = 650 nm, the photoexcitation of **Dim-Mn** induces homolytic cleavage of the Mn-Mn bond to produce two molecules of **Mn**^•^ (Process 3), which recombine to form **Dim-Mn** (Process 2). Thus, a photostationary state exists between **Dim-Mn** and **Mn**^•^ under irradiation at λ_ex_ ≥ 620 nm. Another process in which **Dim-Mn** (*E*_p_^red^ = −1.89 V vs Fc/Fc^+^) participates is reduction by **BI**^•^ (*E*_p_^ox^ = −2.06 V vs Fc/Fc^+^),^64^ which is an exergonic reaction to give the 18-electron species (**TERS-Mn**) and **Mn**^•^ (Process 4). **Dim-Mn** can be reduced only by **BI**^•^ and not by **Os**^**–**^ (*E*_1/2_ = 1.77 V vs Fc/Fc^+^) because of its relatively high reduction potential. Although the steady concentration of **Mn**^•^ should be low during the photocatalytic reaction compared to that of **Dim-Mn**, the direct reduction of **Mn**^•^ to **TERS-Mn** may proceed as a minor process in the photocatalytic reaction. The protonation of **TERS-Mn** produces **Mn-H** (Process 5), which reacts with CO_2_ to generate **Mn-OCOH** (Process 6). In the early stages of the photocatalytic reaction, **Mn-CO**_**2**_**-TEOA** is converted to **Mn-OCOH** as the main Mn complex that formed during the photocatalytic reaction. **Mn-OCOH** accepts one electron from **Os**^**–**^ or **BI**^•^ to induce cleavage of the Mn–O bond to form HCOO^–^ and the 17-electron species **Mn**^•^ (Process 1).

During the photocatalytic reaction using excitation light at λ_ex_ ≥ 480 nm, CO is efficiently released from **Dim-Mn** and induces the decomposition of **Dim-Mn** (Process 7). This was the main deactivation pathway in this photocatalytic system using the excitation light at λ_ex_ ≥ 480 nm.

## Conclusion

In this study, we experimentally and computationally investigated the photochemical reactivities of **Dim-Mn**, which is often formed during photocatalytic reactions in which *fac*-[Mn^I^(bpy)(CO)_3_X]-type complexes are used as catalysts. **Dim-Mn** undergoes two different photoreactions depending on the wavelength of irradiation, i.e., the release of CO by irradiation at λ_ex_ ≥ 480 nm and the homolytic cleavage of the Mn–Mn bond by irradiation at λ_ex_ ≥ 620 nm. This wavelength dependence of the photochemical reactivity of **Dim-Mn** significantly affects the durability of the photocatalytic system using **Mn-CO**_**2**_**-TEOA** as the catalyst, **Os** as the photosensitizer, and **BIH** as the reductant. Although the CO cleavage induces deactivation of the photocatalysis (TON_HCOO–_ < 50) at λ_ex_ ≥ 480 nm, irradiation with longer wavelengths enables photocatalysis with much higher durability (TON_HCOO–_ > 1700 using light at λ_ex_ ≥ 620 nm).

Our *in situ* UV–vis and FT-IR spectroscopic analyses of the photocatalytic systems revealed the reaction mechanism. During this photocatalytic reaction, initiated by the redox photosensitized reaction of **Os** irradiated at λ_ex_ ≥ 620 nm, **Dim-Mn**, **Mn-H**, and **Mn-OCOH** are produced, and a photostationary state is established among them. **Dim-Mn** accepts an electron from **BI**^•^ to become **TERS-Mn**, which is protonated. The produced **Mn-H** reacts with CO_2_ to produce **Mn-OCOH**. The one-electron reduction of **Mn-OCOH** produces both HCOO^–^ and **Mn**^•^, which undergoes dimerization to give **Dim-Mn** again.

Noteworthy, the system using **Os** and **Mn-CO**_**2**_**-TEOA** is one of the durable photocatalytic systems which work using long wavelength visible light up to 700 nm.^61,69,70^ For applying the combination system of the Os-complex photosensitizer and the Mn-complex catalyst to more practical condition such as under sun-light irradiation, protection of the Mn complexes including the Mn dimers against the short-wavelength visible light is essential. One of possible ways is additional usage of another photosensitizer with strong absorption at <500 nm, which can give inner filter effect and protect the decomposition of the Mn dimer.

## Supporting Information Available

Experimental section, UV–vis absorption spectral data, FT-IR spectral data, flow electrolysis results, labeling experiments using ^13^CO_2_, fitting analysis of UV–vis absorption spectra, electrochemical data, and theoretical calculations (PDF)
