# Supplementary material for: Development of a Highly Durable Photocatalytic CO2 Reduction Using a Mn-Complex Catalyst: Application of Selective Photosplitting of a Mn(0)–Mn(0) Bond
Source: J Am Chem Soc. 2025 Feb 10;147(7):6236–48. doi: 10.1021/jacs.4c18366 (PMC11848913; doi:10.1021/jacs.4c18366)
Supplement: Supplementary file 1 — ja4c18366_si_001.pdf [file ja4c18366_si_001.pdf]

Supporting Information

Development of a highly durable photocatalytic CO_2 reduction using a Mn-complex catalyst: application of selective photo-splitting of a Mn(0)-Mn(0) bond.

Hiroki Koizumi,[a]* Yusuke Tamaki,[b] Kei Kamogawa,[c] Marco Nicaso,[c] Yutaka Suzuki,[c] Yasuomi Yamazaki,[d] Hiroyuki Takeda,[e] Osamu Ishitani.[f]*

[a]National Institute of Advanced Industrial Science and Technology (AIST), Tsukuba Central 5, 1-1-1 Higashi, Tsukuba, Ibaraki 305-8565, Japan.

[b] National Institute of Advanced Industrial Science and Technology (AIST), 4-2-1 Nigatake, Miyagino, Sendai, Miyagi 983-8551, Japan.

[c] Department of Chemistry, School of Science, Tokyo Institute of Technology, O-okayama 2-12-1-NE-1, Meguro, Tokyo 152-8550, Japan.

[d]Department of Applied Chemistry, The University of Tokyo, Bunkyo, Tokyo 113-8656, Japan

[e]Division of Molecular Science, Faculty of Science and Technology, Gunma University, 1-5-1 Tenjin, Kiryu, Gunma 376-8515, Japan.

[f]*Department of Chemistry, Graduate School of Advanced Science and Engineering, Hiroshima University, 1-3-1 Kagamiyama, Higashi-Hiroshima, Hiroshima 739 8526, Japan.*

Contents

1. UV-vis and FT-IR spectra of *in-situ* synthesized **Dim-Mn** (Figure S1) ········ S2
2. UV-vis spectral changes of **Dim-Mn** in dark after photoreaction (Figure S2) ········ S2
3. Comparison of UV-vis differential spectrum during photoreaction and UV-vis spectra of **Dim-Mn** (Figure S3) ········ S3
4. Optimize structure of **Dim-Mn** (Figure S4) ········ S3
5. Proposed photoreaction mechanism of **Dim-Mn** (Scheme S1) ········ S4
6. Orbital distribution for Mn center and each ligand of **Dim-Mn** (Table S2) ········ S4
7. GC-MS charts for $HCOO^-$ in the photocatalytic reaction (Figure S5) ········ S5
8. UV-vis differential spectrum of **Os**$^-$ and UV-vis differential spectral changes during photoreaction of **Os** with **BIH** (Figure S6) ········ S5
9. Enlarged FT-IR spectra of Figure 10a at 1950-1880 cm^{-1} (Figure S7) ········ S6
10. FT-IR spectra of the photocatalytic reaction solution before and after Ar bubbling (Figure S8) ········ S6
11. FT-IR spectral changes measured immediately after the photocatalytic reaction (Figure S9) ········ S7
12. UV-vis spectra of **Mn-CO_2-TEOA** and **Mn-OCOH** (Figure S10) ········ S7
13. FT-IR spectra of *in-situ* synthesized **Mn-H** (Figure S11) ········ S8
14. Method of the subtraction to obtain UV-vis spectra of **Mn-H** (Figure S12) ········ S9
15. Fitting results for observed UV-vis spectra during the reaction of **Mn-H** with CO_2 (Figure S13) ········ S10
16. Table S2. The summary of electrochemical data of related compounds. S10
17. Experimental section ········ S10
18. References ········ S13

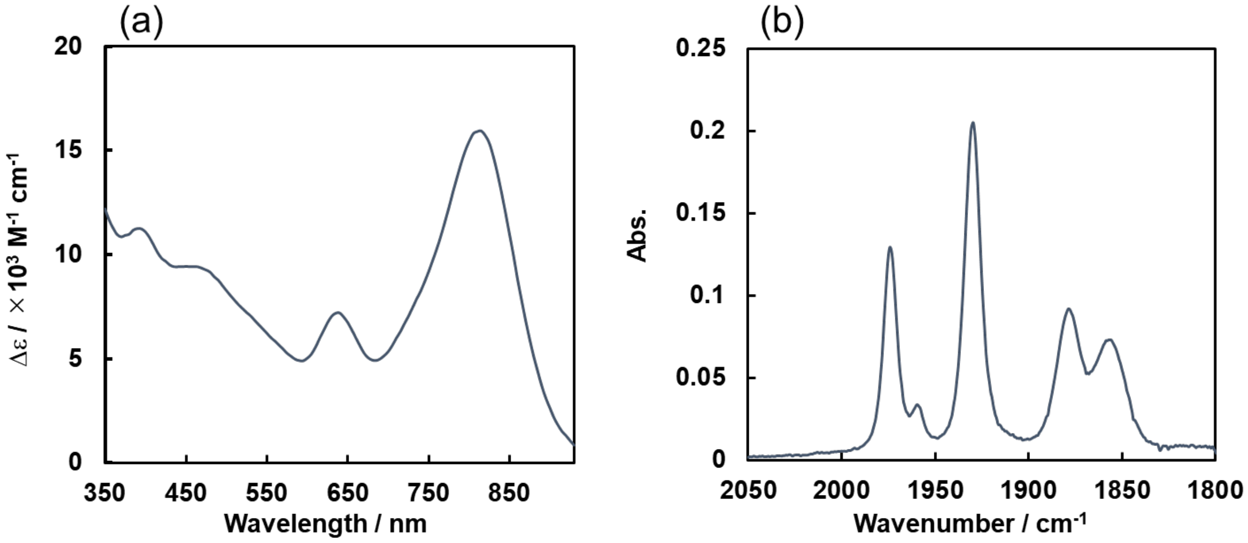

Figure S1. UV-vis and FT-IR spectra of *in-situ* synthesized **Dim-Mn**. The UV-vis absorption spectrum was obtained by the subtraction of the spectrum of **[CoCp*]**$^+$ from the observed spectrum. These spectra were measured in DMA-TEOA (5:1, v/v) containing 0.5 mM of **Dim-Mn**.

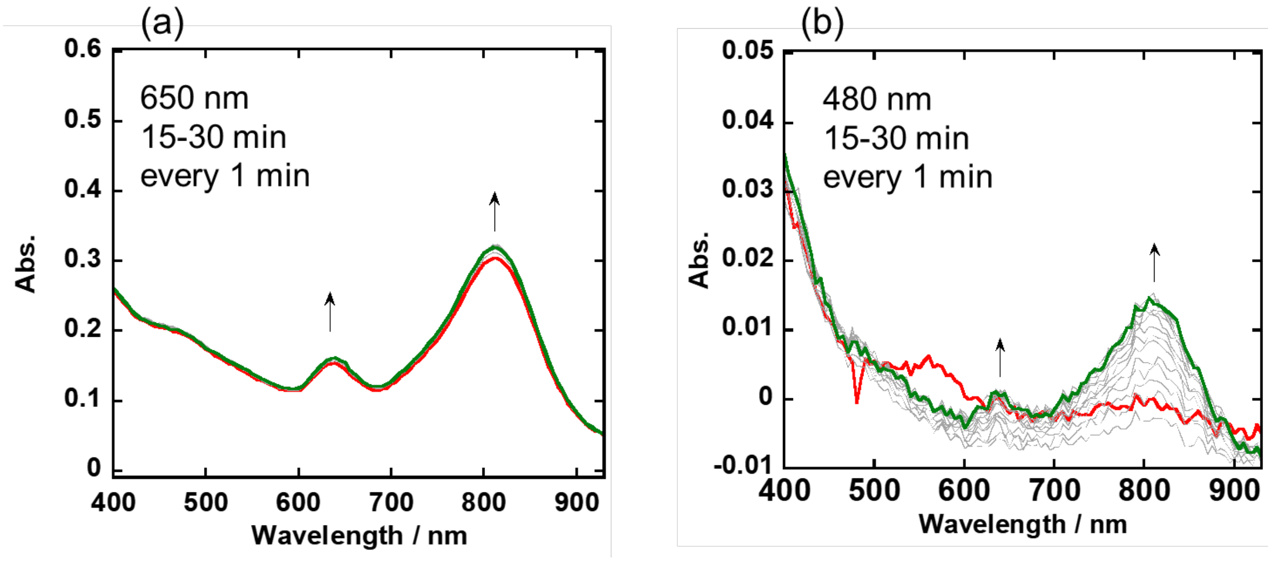

Figure S2. UV-vis spectral changes of **Dim-Mn** in dark after photoreaction. (a) λ_{ex} = 650 nm, (b) λ_{ex} = 480 nm. Each photoreaction was carried out for 15 min irradiation in DMA-TEOA (5:1, v/v).

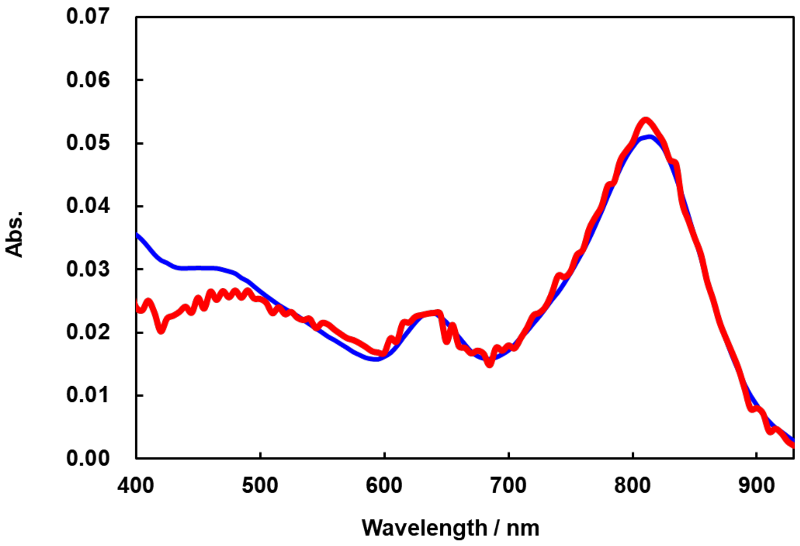

Figure S3. Comparison of UV-vis differential spectrum during photoreaction (red line) and UV-vis spectra of Dim-Mn (blue line). The red line spectrum was obtained by subtracting the spectrum during photoreaction at 15 min under $\lambda_{\varepsilon x}$ = 650 nm light (Figure 2a, red line) from the spectra in the dark for 15 min after the photoreaction (Figure S2a, green line).

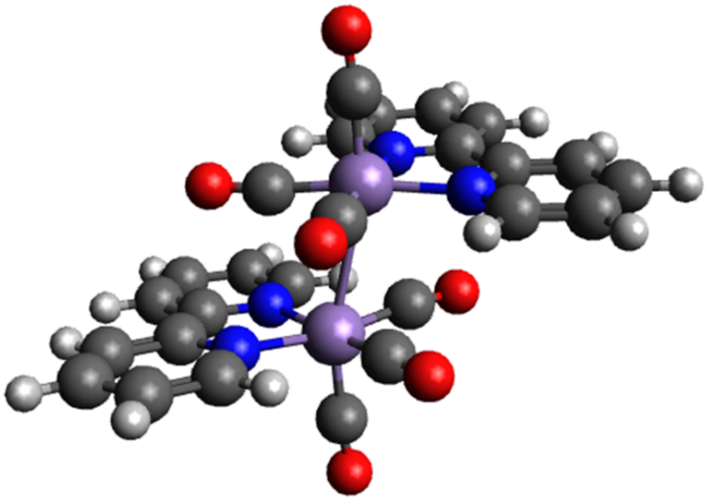

Figure S4. Optimized structure of **Dim-Mn**.

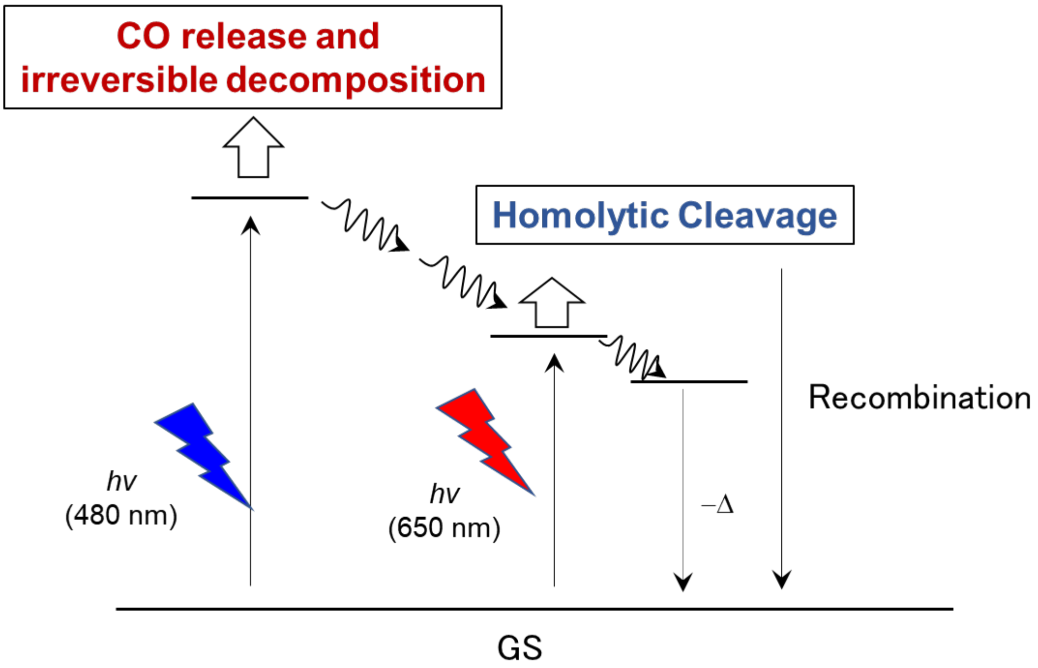

Scheme S1. Proposed photochemical reaction mechanism of **Dim-Mn**.

Table S1. Orbital distribution for Mn center and each ligand of **Dim-Mn**.

Orbital	Energy / eV	Orbital distribution / %		
		bpy	Mn	CO
LUMO+7	-0.21	14	34	52
LUMO+6	-0.22	6	46	47
LUMO+5	-0.90	96	0	4
LUMO+4	-0.97	94	2	3
LUMO+3	-1.1	96	2	1
LUMO+2	-1.14	92	6	2
LUMO+1	-1.74	82	14	4
LUMO	-2.15	94	4	2
HOMO	-4.53	32	54	16
HOMO-1	-6.0	6	70	24

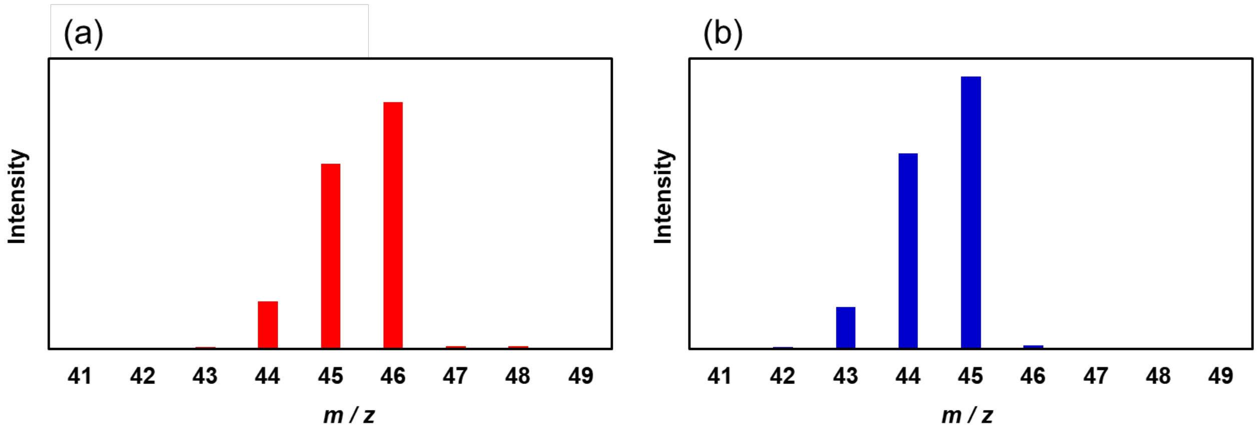

Figure S5. GC-MS charts for $HCOO^-$ in the photocatalytic reactions. Photocatalytic reactions were conducted under $^{13}CO_2$ (a) or CO_2 (b), respectively.

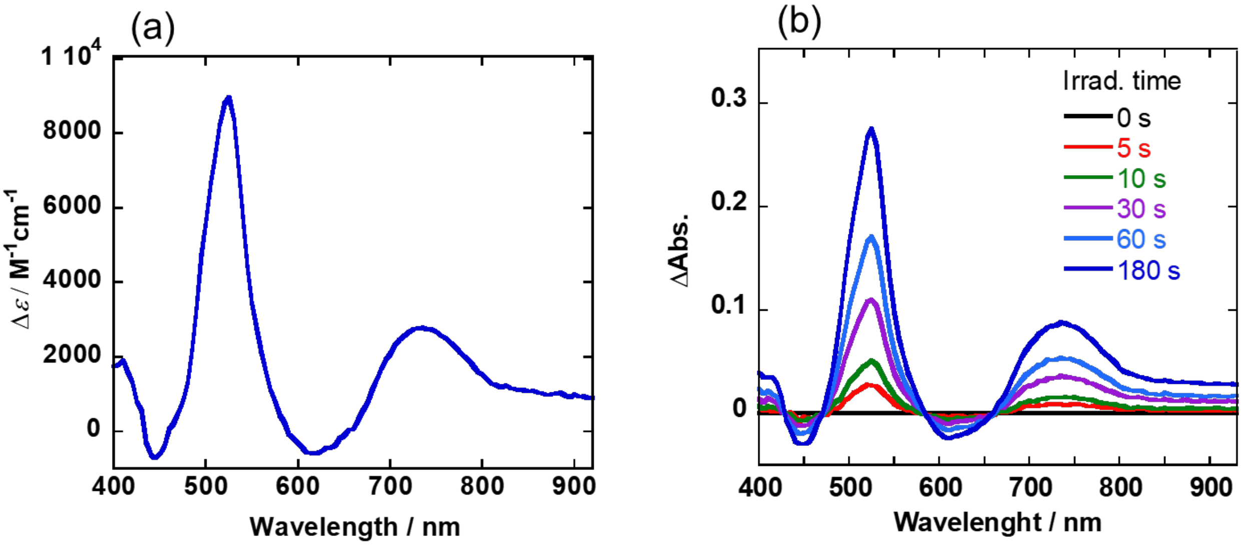

Figure S6. (a) UV-vis differential spectrum of **Os**$^-$ and (b) UV-vis differential spectral changes during photoreaction of **Os** with **BIH**. The UV-vis differential spectrum was obtained by subtracting the UV-vis spectrum of **Os** from observed spectrum in flow electrolysis of **Os**. UV-vis differential spectral changes were obtained by subtracting the spectrum before photoreaction from those recorded during photoreaction. Photoreaction was conducted in DMA-TEOA (5:1 v/v) containing 0.05 mM of **Os** with 0.1 M of **BIH** under λ_{ex} = 650 nm.

Immediately after starting irradiation, absorptions at approximately 520 nm and ~740 nm increased. These absorptions were attributed to **Os**$^-$,as confirmed by comparison with Figure S6a.

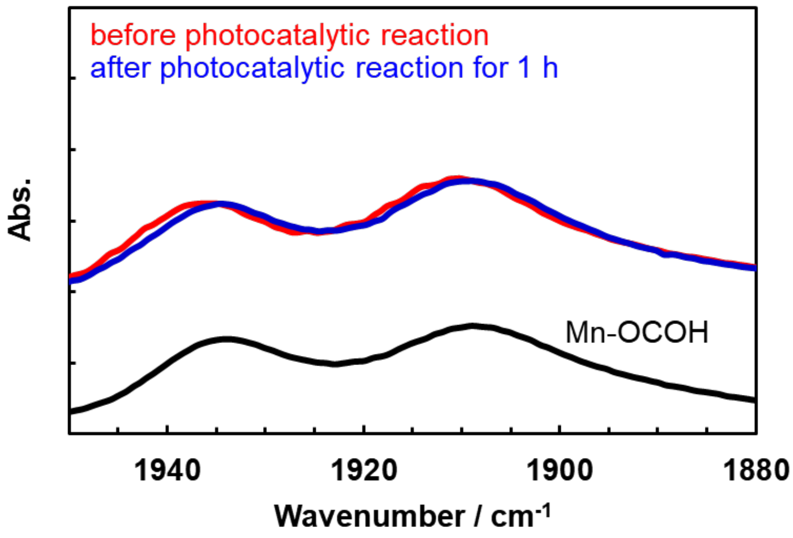

Figure S7 Enlarged FT-IR spectra of Figure 10a at 1950–1880 cm^{-1}.

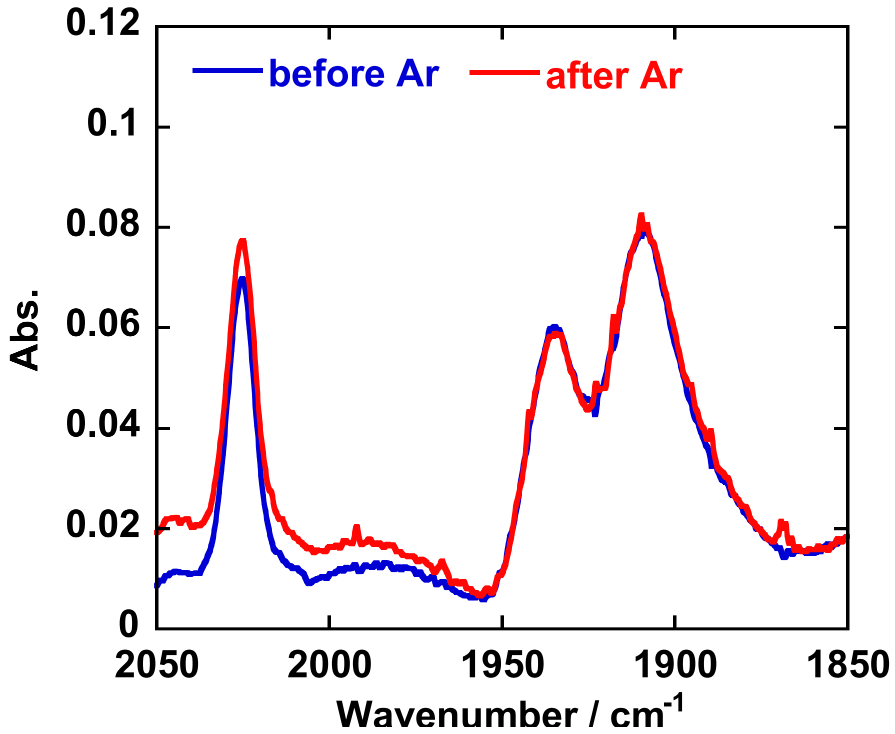

Figure S8. FT-IR spectra of the photocatalytic reaction solution before and after Ar bubbling.

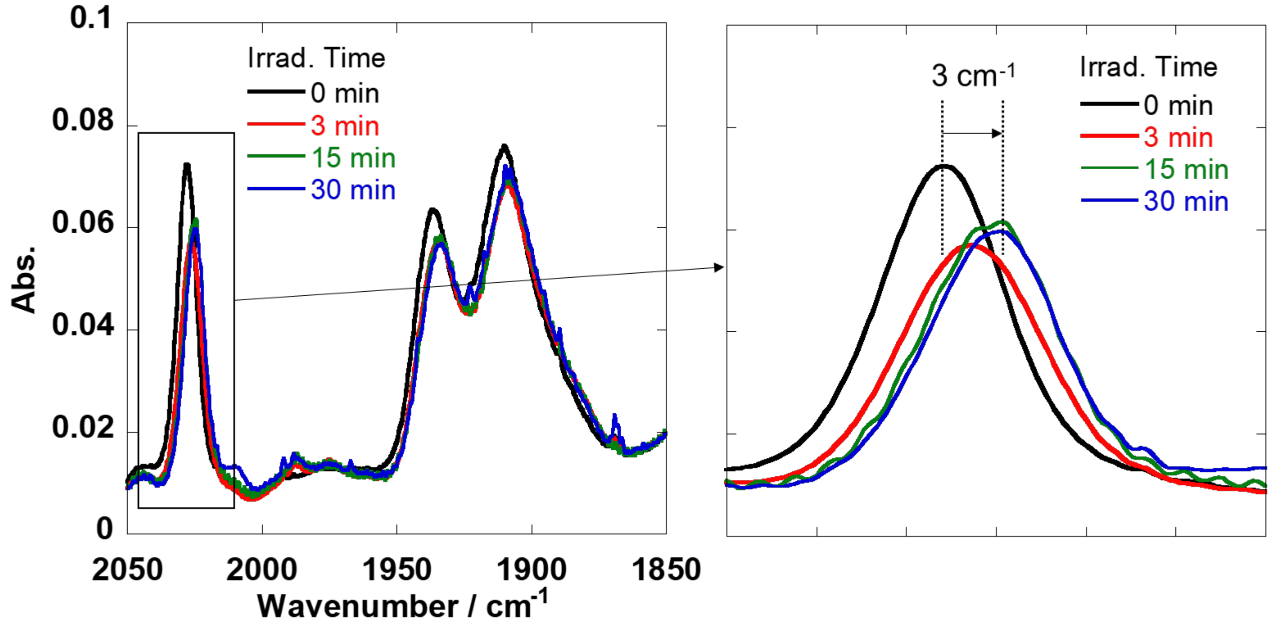

Figure S9. FT-IR spectral changes measured immediately after the photocatalytic reactions. Photocatalytic reactions were conducted in a DMA-TEOA (5;1 v/v) solution containing **Mn-CO_2-TEOA** (0.5 mM), **Os** (0.05 mM), and **BIH** (0.1 M) under CO_2 with irradiation at $\lambda_{ex} \geq 640$ nm in an IR cell.

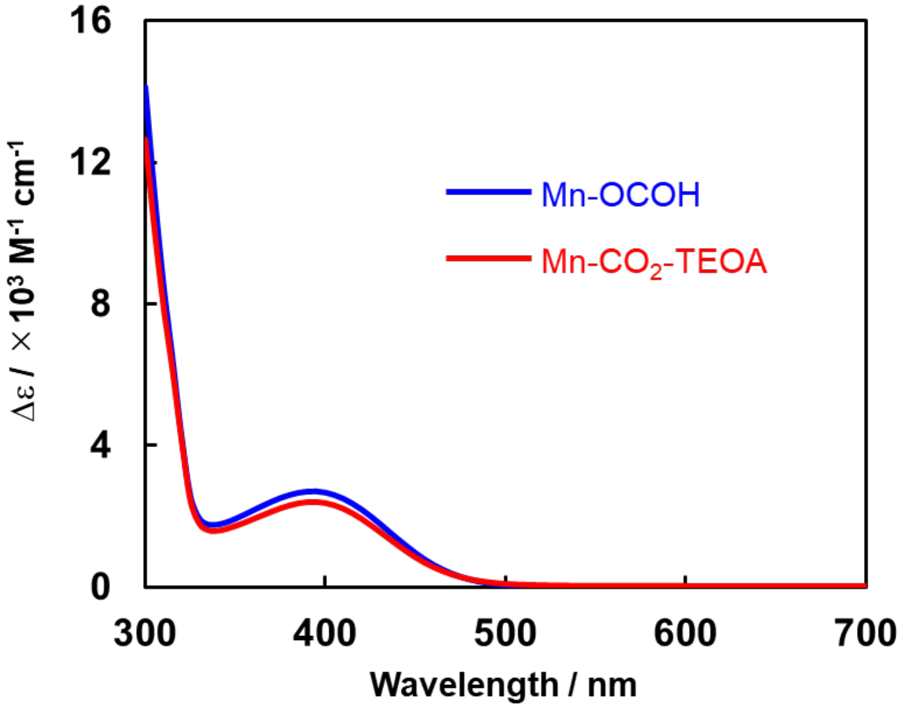

Figure S10. UV-vis spectra of **Mn-CO_2-TEOA** and **Mn-OCOH**. These spectra were measured in DMA-TEOA (5:1 v/v).

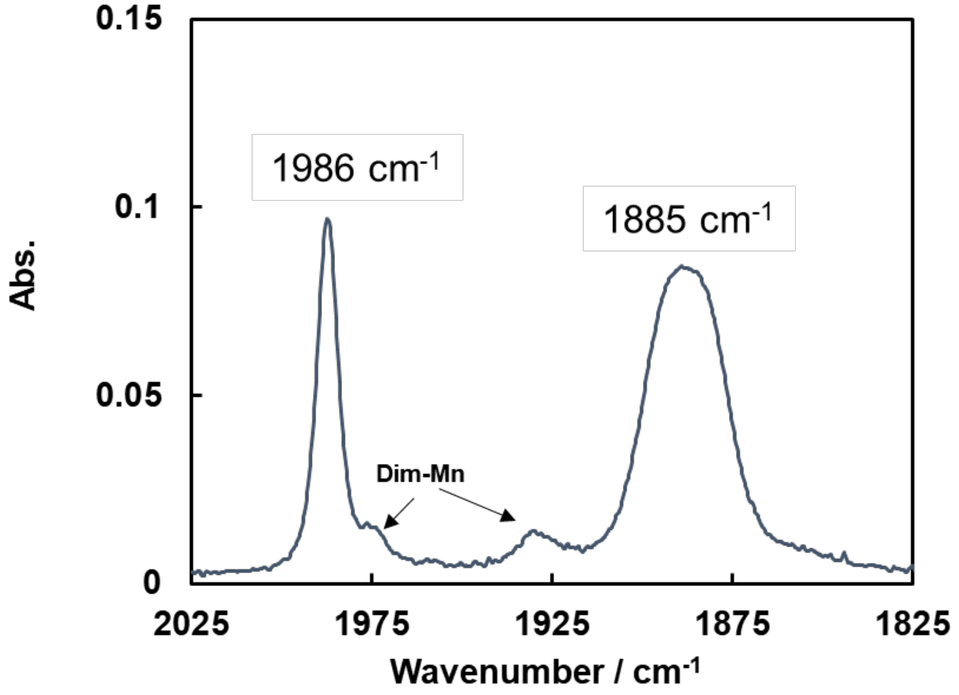

Figure S11. FT-IR spectra of *in-situ* synthesized **Mn-H**. This spectrum was obtained in DMA-TEOA (5:1 v/v) containing a small amount of **Dim-Mn**.

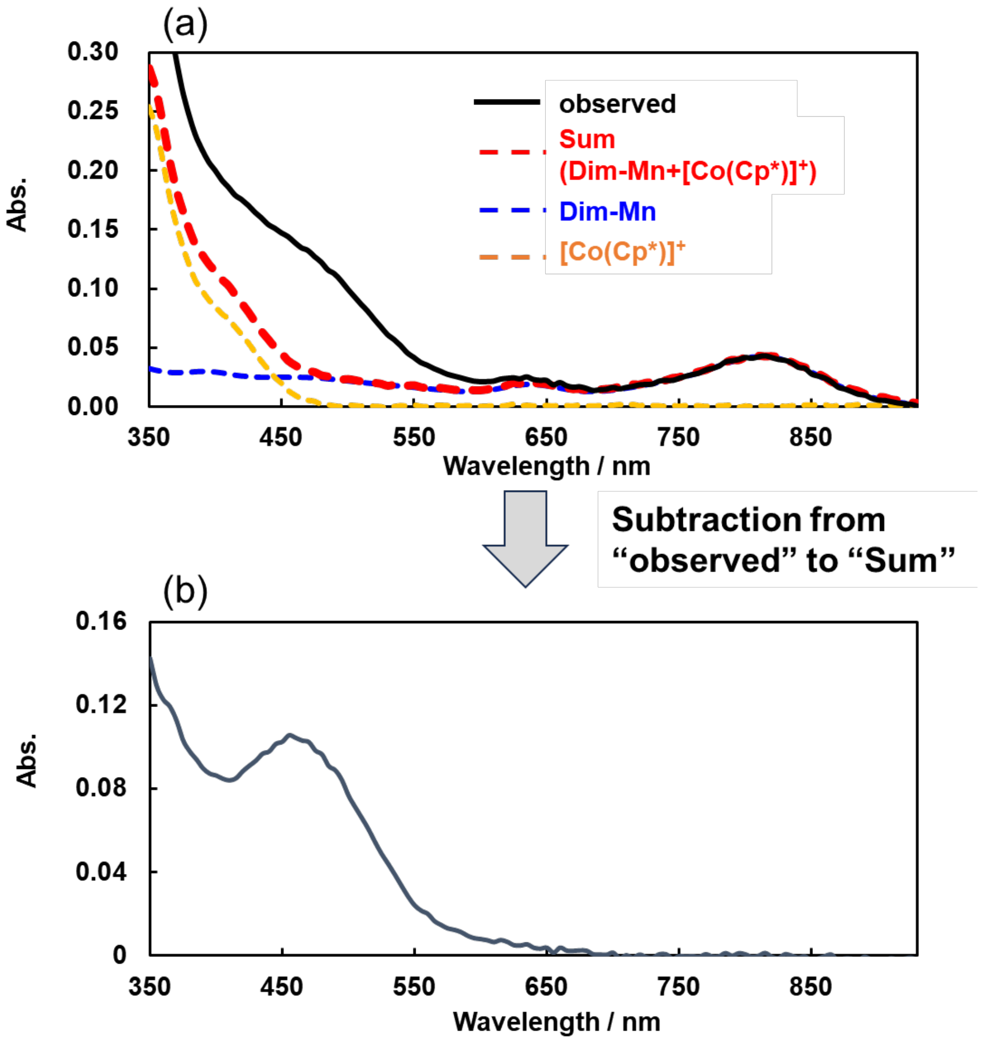

Figure S12. Method of the subtraction to obtain UV-vis spectra of **Mn-H**. (a) observed UV-vis absorption spectrum of a DMA-TEOA (5:1, v/v) solution containing *in-situ* synthesized **Mn-H** (black line), spectra of **Dim-Mn** (blue dashed line) and **[CoCp*]**$^+$ (orange dashed line), and sum of the spectra of **Dim-Mn** and **[CoCp*]**$^+$ (red dashed line). (b) Obtained UV-vis absorption spectrum of *in-situ* synthesized **Mn-H** by subtracting the red dashed line from the black line. The DMA-TEOA (5:1, v/v) solution containing **Mn-H** was prepared by mixing **Mn-Br** (0.50 mM), **CoCp*** (2.0 mM), followed by the addition of NH_4PF_6 (5 mM).

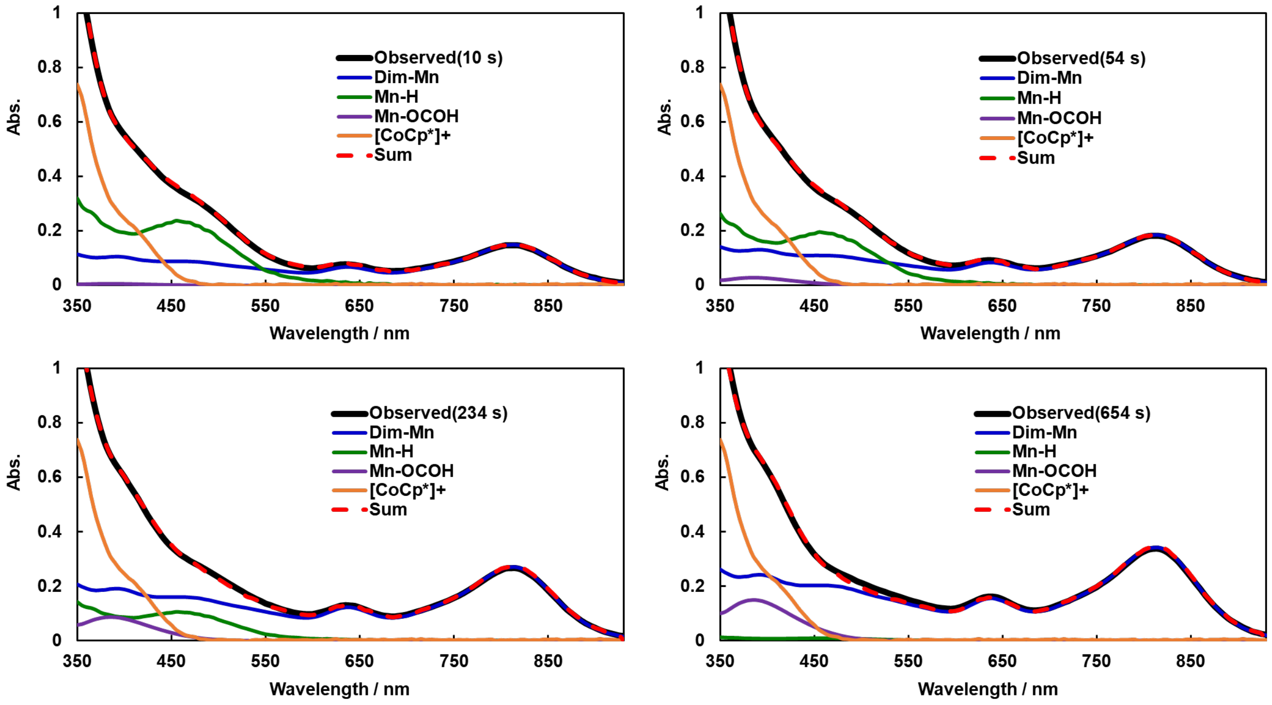

Figure S13. Fitting results for observed UV-vis spectra during the reaction of **Mn-H** with CO_2. These observed spectra during the reaction were measured in DMA-TEOA (5:1 v/v).

Table S2. The summary of electrochemical data of related compounds.

Complex	E_p^{red} vs. Fc/Fc$^+$	E_p^{ox} vs. Fc/ Fc$^+$	$E_{1/2}$ vs. Fc/Fc$^+$
**Mn-CO₂-TEOA**	-1.79	-	-
**Dim-Mn**[a]	-1.89	-	-
**BI**$^\bullet$	-	-2.06[b]	-
**Os**	-		-1.77 (ΔE = 82 mV)

a: Measured by using **Mn-Br** under Ar. b: reported in the reference S2.

Experimental section

General procedures. UV-vis spectra were measured with a JASCO V-565 spectrometer. IR spectra were recorded using a JASCO FT/IR-6600 spectrometer with a JASCO fixed cell for liquid with CaF_2 window (L = 0.5 mm) at 1 cm^{-1} resolution. ^{1}H-NMR and ^{13}C-NMR spectra were measured in acetonitrile-d_3 or DMSO-d_6 using a JEOL ECA400-II (400 MHz) system. Electrospray ionization mass spectroscopy (ESI-MS) was performed using a SHIMADZU LCMS-2010A system with acetonitrile as a mobile phase. Cyclic voltammograms of the Mn(I) complexes and **Os** were recorded in DMA-TEOA mixed solutions containing the complex (0.5 mM) and Et_4NBF_4 (0.1 M) as a supporting electrolyte employing ALS/CHI BAS CHI-720 (BAS) as a potentiostat with a glassy carbon as a working electrode, a Pt wire as counter electrode, and Ag/AgNO$_3$ (0.01 M) as a reference electrode. Obtained potentials were corrected from vs. Ag/AgNO$_3$ to vs. Fc/Fc$^+$ using $E_{1/2}$ of Ferrocene (0.09 V vs. Ag/AgNO$_3$) in this solution The gaseous reaction products, CO and H_2, were analyzed by GC-TCD (GL science, GC323) with Ar as carrier gas and

an active carbon column. $HCOO^-$ was analyzed by a capillary electrophoresis system (AGILENT TECHNOLOGE, 7000L Capillary Electrophoresis or OTUKA ELECTRONICS CAPI-3300I).

Materials. DMA was distilled under reduce pressure after pre-drying by activated molecular sieves 4A over 3 days. TEOA was distilled under reduced pressure and stored in a glove box filled by Ar. Other reagents in reagent–grade quality were used without further purification. Ar and CO_2 (CO_2 conc.: 99.99%) were purchased from Iwatani.

Synthesis. **Mn-Br**, *fac*-[Mn(bpy)$(CO)_3$(MeCN)] (PF_6), **Os**, **BIH** and **[CoCp*]$^+$** were synthesized according to the literature methods.[1-4]

Mn-OCOH. **Mn-Br** (0.11 g, 0.30 mmol) and NaOC(O)H (0.73 g, 11 mmol) were dissolved in a Ar saturated $MeOH/H_2O$ (1:1 v/v) mixed solution and stirred at 80 °C under Ar for overnight. After cooling this reaction mixture to room temperature, MeOH was removed under reduced pressure by evaporation, precipitated yellow solid were taken by filtration. This solid was dissolved in CH_2Cl_2 and washed with water (30 mL × 3). The organic phase was dried using Na_2SO_4, then the solvent was removed by evaporation. **Mn-OCOH** was obtained as yellow solid after recrystallization with acetone/hexane three times. Yield: 25 mg (25 %). ^{1}H NMR (400 MHz, acetonitrile-d_6, ppm): d = 9.22 (d, 2H, J = 6.2 Hz, bpy-6,6'), 8.72 (d, 2H, J = 8.0 Hz, bpy-3,3'), 8.25 (dd, 2H, J = 6.6, 8.0 Hz, bpy-5,5'), 8.03 (s, H, OC(O)-H), 7.73 (dd, 2H, J = 6.2, 6.6 Hz, bpy-5,5'). FT-IR (DMA-TEOA): n_{CO} / cm^{-1}, 2025, 1934, 1910.

Preparation of the following Mn complexes and measuring their spectra except sonication for dissolving **Co(Cp*)** were conducted in a glove box (UNICO, UN-650F) with a gas circulation dehydration device (DGE-05) under Ar (water content was less than 376 ppm). The front side panel of the glove box was covered by red film to avoid photochemical decomposition of the Mn complexes.

Dim-Mn. 8.2 mg of **Co(Cp*)** was dissolved by sonication in 5 mL of DMA-TEOA (5:1 v/v) in a tightly sealed vial. This solution was added to a DMA-TEOA (5:1 v/v) solution containing 5.0 mM of **Mn-Br** and then this mixture was diluted with additional 5 mL of DMA-TEOA. The volume of the **Co(Cp*)** solution was appropriately adjusted to form 0.5 mM of **Dim-Mn** by careful check by using FT-IR spectroscopy because determination of the concentration of **Co(Cp*)** was difficult due to its rapid oxidation by contaminated oxygen. In addition, we carefully checked that **Co(Cp*)** was completely consumed because an excess amount of **Co(Cp*)** induced formation of **TERS-Mn**. The UV-vis absorption spectrum was recorded using a high sensitivity multichannel photodetector (OTSUKA Electronics, MCPD-9800) and a D_2/I_2 (OTSUKA Electronics, MC-2530) lamp. The molar coefficient (e) of **Dim-Mn** was calculated by subtraction of the UV-vis spectrum of **[CoCp*]$^+$** from the observed spectrum, which is the oxidized **Co(Cp*)** by reduction of **Mn-Br**. Concentration of **[CoCp*]$^+$** was equaled to the amount of **Co(Cp*)** added after the reaction.

Mn-H. 8.2 mg of **Co(Cp*)** was dissolved in 5 mL of DMA-TEOA (5:1 v/v) by sonication in a tightly sealed vial. This solution was added to 0.5 mL of a DMA-TEOA (5:1 v/v) solution containing 5.0 mM of **Mn-Br** or 50 mM of NH_4PF_6 as a proton source, and this mixture was then diluted with 5 mL of DMA-TEOA. The volume of the **Co(Cp*)** solution was adjusted to leave a small amount of **Dim-Mn** to avoid remaining excess **Co(Cp*)** by careful checking by FT-IR spectroscopy. The UV-vis spectrum was recorded in a tightly sealed quartz cell (L = 0.1 cm) using the MCPD-9800 detector and the D_2/I_2 lamp. The e value of **Mn-H** was calculated by subtraction of the UV-vis spectra

of **[CoCp*]**$^+$and **Dim-Mn** from the observed UV-vis spectrum as shown in Figure S12. The observed concentration of **[CoCp*]**$^+$ was equaled to the added amount of **Co(Cp*)** after the reaction.

Photochemical reaction of Dim-Mn. A **Dim-Mn** solution was prepared as described above. This solution was irradiated by a 500 W Xe lamp with a 650 nm or 480 nm bandpass filter (FWHM: 10 nm) in a lamp-house irradiation apparatus with a tightly sealed quartz cell (L = 1 cm). UV-vis absorption spectra during the photochemical reactions were recorded using MCPD-9800) and the D_2/I_2 lamp.

DFT calculation. DFT and TD-DFT calculations were carried out using Gaussian 16 packages.[5] The geometry optimization for **Dim-Mn** were carried out using PBE1PBE/def2-SVP with solvent effect of DMA, which was modelled as a dielectric continuum using a polarizable continuum model. The contribution of the central metal and ligands to each molecular orbital was calculated using GaussSum[6] based on the Mulliken electron density analysis. TD-DFT excited state calculations to obtain oscillator strength were conducted by PBE1PBE/def2-SVP in the DMA solvent as a dielectric continuum, using the structure optimized by DFT calculation.

Photocatalytic reaction. All of sample preparations were operated in the glove box (UNICO, UN-650F). The front side panel of the glove box was covered by red film to avoid decomposition of the Mn complexes.

As a typical photocatalytic reaction, a 4 mL DMA-TEOA (5:1 v/v) solution containing 0.05 mM of **Os**, 0.05 mM of **Mn-CO$_2$-TEOA** and 0.1 M of **BIH** in a Pyrex test tubes (i.d. = 8 mm, 11 mL) was bubbled with CO_2 for 30 min and this solution was irradiated in an merry-go-round irradiation apparatus (Eikosha) with a 300 W halogen lamp with a rhodamine or K_2CrO_7 aqueous solution filter (d = 1 cm) for selecting light at ⩾ 480 nm or ⩾ 620 nm, respectively. The temperature of the solutions was controlled at 25 °C by a constant temperature system (EYELA CTP1000) during photocatalytic reaction.

Photocatalytic reactions for obtaining quantum yields and *in-situ* UV-vis spectrum measurements were conducted in a lamp-house irradiation apparatus. A same solution as described above was purged with CO_2 for 30 min by bubbling in a quartz cubic tube (L = 1 cm) and this solution was irradiated by using a 500 W Xe lamp and a corresponding band pass filter (FWHM: 10 nm). The temperature of the solutions was controlled at 25±0.1 °C by a constant temperature system (IWAKI, CTS-134A). Light intensity of the Xe lamp was determined by chemical actinometry using $K_2[Fe(C_2O_4)_3]$. *In-situ* UV-vis absorption spectra were recorded by the MCPD-9800 detector and the D_2/I_2 lamps.

Photocatalytic reactions to obtain quasi *in-situ* FT-IR spectra were conducted using a FT-IR optical cell (JASCO fixed cell for liquid with CaF_2 window (L = 0.5 mm)). A DMA-TEOA (5:1 v/v) containing **Os** as the photosensitizer (0.5 mM), **Mn-CO$_2$-TEOA** as a catalyst (0.5 mM) and BIH (0.1 M) was bubbled with CO_2 for 30 min and transferred to the FT-IR optical cell. This solution was irradiated by a 500W Xe lamp with a rhodamine aqueous solution filter (d = 5 cm, ⩾ 620 nm). After the irradiation, Measurement of FT-IR spectra started within 10 s.

$^{13}CO_2$ labeling experiments. A DMA-TEOA (5:1 v/v) solution containing 1.0 mM of **Os**, 1.0 mM **Mn-CO$_2$-TEOA** and 0.1 M of **BIH** was deaerated three times via a freeze–pump–thaw cycles and then added $^{13}CO_2$ (630 mmHg). This solution was irradiated with a LED lamp (λ_{ex} = 620 nm) for 19 h. Formic acid was extracted with ethyl acetate prior to the GC-MS analyses using the reported method.[7] The ethyl acetate phase was analyzed by using Shimadzu GCMS-QP2010 equipped with a SH-1 column (i.d. 0.32 mm, 60.0 m).

Flow electrolysis. Flow electrolysis was carried out using the ALS/CHI BAS CHI-720 potentiostat with a flow-electrolysis cell (VF-2, EC Frontier) combining a carbon felt as a working electrode, a Pt wire counter electrode, 0.01 M of Ag/$AgNO_3$ as a reference electrode. UV-vis absorption spectra after the reduction were measured in a quartz cell (L = 1.5 mm) connected with the flow-electrolysis cell by using the MCPD-8500 detector and the D_2/I_2 lamp. A DMA solution containing 0.5 mM of **Os** and 0.1 M of Et_4NBF_4 as the electrolyte was bubbled with Ar bubbling for 30 min and pumped into VF-2. Various voltages were applied to the electrodes for obtaining a voltage-current curve, and the potential was kept until the current value and spectrum are stabilized. Number of electrons used for reduction of **Os** was estimated by obtaining number of flowed electrons at the potential and the following equation:

$$\text{Number of electrons (n)} = \frac{i - i_b}{\text{CFv}}$$

where i is the observed current value, i_b is the current value without the substrates, C is the concentration of **Os**, F is the Faraday constant (C mol^{-1}), and v was the flow rate.

Reaction of Mn-H with CO_2. According to the described method, a DMA-TEOA solution (5:1 v/v) containing 0.11 mM of **Mn-H** and 0.01 mM of **Dim-Mn** was prepared in a tightly sealed quartz cell (L = 1.0 cm). 0.2 mL of a CO_2 saturated DMA solution was injected into this solution using a gas-tight syringe. UV-vis spectra after this addition were recorded by using the MCPD-9800 and the D_2/I_2 lamp. The obtained UV-vis spectra were fitted with the corresponding spectra of **Mn-H**, **Dim-Mn**, **Mn-OCOH** and **[CoCp*]**$^+$.

References

(S1) Koizumi, H.; Chiba, H.; Sugihara, A.; Iwamura, M.; Nozaki, K.; Ishitani, O. CO_2 capture by Mn(I) and Re(I) complexes with a deprotonated triethanolamine ligand. *Chem. Sci.* **2019**, *10* (10), 3080-3088, DOI: 10.1039/C8SC04389B.

(S2) Tamaki, Y.; Koike, K.; Morimoto, T.; Yamazaki, Y.; Ishitani, O. Red-Light-Driven Photocatalytic Reduction of CO_2 using Os(II)–Re(I) Supramolecular Complexes. *Inorg. Chem.* **2013**, *52* (20), 11902-11909. DOI: 10.1021/ic4015543.

(S3) Chikashita, H.; Itoh, K. $AlCl_3$-Promoted Conjugate Reduction of α,β Unsaturated Carbonyl Compounds with 1,3-Dimethyl-2-phenylbenzimidazoline. *Bull. Chem. Soc. Jpn.* **2006**, *59* (6), 1747-1752. DOI: 10.1246/bcsj.59.1747.

(S4) Robbins, J. L.; Edelstein, N.; Spencer, B.; Smart, J. C. Syntheses and electronic structures of decamethylmetallocenes. *J. Am. Chem. Soc.* **1982**, *104* (7), 1882-1893. DOI: 10.1021/ja00371a017.

(S5) Frisch, M. J.; Trucks, G. W.; Schlegel, H. B.; Scuseria, G. E.; Robb, M. A.; Cheeseman, J. R.; Scalmani, G.; Barone, V.; Petersson, G. A.; Nakatsuji, H.; Li, X.; Caricato, M.; Marenich, A. V.; Bloino, J.; Janesko, B. G.; Gomperts, R.; Mennucci, B.; Hratchian, H. P.; Ortiz, J. V.; Izmaylov, A. F.; Sonnenberg, J. L.; Williams-Young, D.; Ding, F.; Lipparini, F.; Egidi, F.; Goings, J.; Peng, B.; Petrone, A.; Henderson, T.; Ranasinghe, D.; Zakrzewski, V. G.; Gao, J.; Rega, N.; Zheng, G.; Liang, W.; Hada, M.; Ehara, M.; Toyota, K.; Fukuda, R.; Hasegawa, J.; Ishida, M.; Nakajima, T.; Honda, Y.; Kitao, O.; Nakai, H.; Vreven, T.; Throssell, K.; Montgomery, J. A., Jr.; Peralta, J. E.; Ogliaro, F.; Bearpark, M. J.; Heyd, J. J.; Brothers, E. N.; Kudin, K. N.; Staroverov, V. N.; Keith, T. A.; Kobayashi, R.; Normand, J.; Raghavachari, K.; Rendell, A. P.; Burant, J. C.; Iyengar, S. S.; Tomasi, J.; Cossi, M.; Millam, J. M.; Klene, M.; Adamo, C.; Cammi, R.; Ochterski, J. W.; Martin, R. L.; Morokuma, K.; Farkas, O.; Foresman, J. B.; Fox, D. J. *Gaussian 16 Rev. C.01*; Wallingford, CT, 2016.

(S6) O'boyle, N. M.; Tenderholt, A. L.; Langner, K. M. cclib: A library for package-independent computational chemistry

algorithms. *J. Comput. Chem.* **2008**, *29* (5), 839-845. DOI: 10.1002/jcc.20823.

(S7) Bassan, E.; Inoue, R.; Fabry, D.; Calogero, F.; Potenti, S.; Gualandi, A.; Cozzi, P. G.; Kamogawa, K.; Ceroni, P.; Tamaki, Y.; et al. Visible-light driven photocatalytic CO_2 reduction promoted by organic photosensitizers and a Mn(I) catalyst. *Sustain. Energ. Fuels* **2023**, *7* (14), 3454-3463, 10.1039/D3SE00546A. DOI: 10.1039/D3SE00546A.
